# Subnanometer high-entropy alloy nanowires enable remarkable hydrogen oxidation catalysis

**DOI:** 10.1038/s41467-021-26425-2

**Published:** 2021-10-29

**Authors:** Changhong Zhan, Yong Xu, Lingzheng Bu, Huaze Zhu, Yonggang Feng, Tang Yang, Ying Zhang, Zhiqing Yang, Bolong Huang, Qi Shao, Xiaoqing Huang

**Affiliations:** 1 State Key Laboratory of Physical Chemistry of Solid Surfaces, College of Chemistry and Chemical Engineering, Xiamen University, 361005 Xiamen, China; 2 Guangzhou Key Laboratory of Low-Dimensional Materials and Energy Storage Devices, Collaborative Innovation Center of Advanced Energy Materials, School of Materials and Energy, Guangdong University of Technology, 510006 Guangzhou, China; 3 Shenyang National Laboratory for Materials Science, Institute of Metal Research, Chinese Academy of Sciences, 110016 Shenyang, China; 4 College of Chemistry, Chemical Engineering and Materials Science, Soochow University, 215123 Suzhou, China; 5 Department of Applied Biology and Chemical Technology, The Hong Kong Polytechnic University, Hung Hom, Kowloon, Hong Kong SAR China

**Keywords:** Electrocatalysis, Electrocatalysis, Nanowires

## Abstract

High-entropy alloys (HEAs) with unique physicochemical properties have attracted tremendous attention in many fields, yet the precise control on dimension and morphology at atomic level remains formidable challenges. Herein, we synthesize unique PtRuNiCoFeMo HEA subnanometer nanowires (SNWs) for alkaline hydrogen oxidation reaction (HOR). The mass and specific activities of HEA SNWs/C reach 6.75 A mg_Pt+Ru_^−1^ and 8.96 mA cm^−2^, respectively, which are 2.8/2.6, 4.1/2.4, and 19.8/18.7 times higher than those of HEA NPs/C, commercial PtRu/C and Pt/C, respectively. It can even display enhanced resistance to CO poisoning during HOR in the presence of 1000 ppm CO. Density functional theory calculations reveal that the strong interactions between different metal sites in HEA SNWs can greatly regulate the binding strength of proton and hydroxyl, and therefore enhances the HOR activity. This work not only provides a viable synthetic route for the fabrication of Pt-based HEA subnano/nano materials, but also promotes the fundamental researches on catalysis and beyond.

## Abstract (web summary)

High-entropy alloys (HEAs) have attracted increasing attention in diverse field. Here, the authors report PtRuNiCoFeMo HEA with enhanced activity, stability and preferable CO anti-poisoning in alkaline hydrogen oxidation reaction.

## Introduction

Hydrogen fuel cell is considered as one of the most promising energy conversion devices due to its high energy efficiency and pollution-free feature^1–5^. Over the past decades, great efforts have been devoted to developing catalysts for hydrogen oxidation reaction (HOR) for enhancing the energy conversion efficiency of the hydrogen fuel cell^6,7^. The ideal catalysts for HOR should simultaneously fulfill the features of high activity, high durability as well as low cost. As the state-of-the-art catalyst, PtRu-based alloys have been widely used for HOR in recent years^8,9^. Despite great progress has been achieved, PtRu-based catalysts still suffer from the drawbacks, including high-cost, poor stability, and low resistance to CO-poisoning. It is thus highly desired to develop highly efficient catalysts for HOR under operating conditions.

Recently, high-entropy alloys (HEAs) have attracted increasing attention in diverse fields due to their inherent properties, including high thermal stability, strong mechanical strength, excellent corrosion resistance, and fine trade-off effect on performance^10–12^. As promising catalysts in heterogeneous catalysis, HEAs generally consist of five or more metals, which display the structure-dependent synergistic effects and enhanced catalytic performance in terms of activity and durability^13,14^. Moreover, since the HEAs can largely decrease the usage of noble metals and thus reduce the cost of catalyst^15^, they have been attracted increasing research interests. For instance, Li et al.^16^ reported a small HEAs Pt_18_Ni_26_Fe_15_Co_14_Cu_27_ nanoparticles (NPs) with a mean size of ~3.4 nm as an efficient catalyst for alkaline methanol oxidation reaction, on which the synergistic effects facilitate the site-to-site electron transfer during both reduction and oxidation processes. Cui et al.^17^ demonstrated that the synergistic effects between different metals in CrMnFeCoNi HEA sulfide can regulate the electronic states and thus enhances the oxygen evolution reaction activity. Despite the great potentials of HEAs, the controlled synthesis of PtRu-based HEAs with tailored morphologies and sizes still remains great challenges, which severely impedes their applications. To this end, it is highly desirable to develop facile strategies for fabricating PtRu-based HEAs.

Herein, we fabricated PtRuNiCoFeMo HEA subnanometer nanowires (SNWs) as highly active and durable catalyst for HOR. In particular, the mass activity of HEA SNWs for HOR is 4.1 and 19.8 times higher than that of commercial PtRu/C and Pt/C, respectively. Moreover, no obvious decay of HOR performance was observed after 2000 cycles in the accelerated durability test (ADT), suggesting the promising stability of HEA SNWs for HOR. Additionally, HEA SNWs can even display superior resistance to CO poisoning comparing to commercial PtRu/C and Pt/C in the presence of 1000 ppm CO. Density functional theory (DFT) calculations confirm that the strong interactions between different metals in HEA SNWs can regulate the electronic structures of different metals and thus enhance the HOR activity. In particular, Co and Ni sites maintain the highly stable valence states due to the pinning effect by nearby Fe and Mo sites, and the Pt and Ru sites modulate the overall electroactivity for superior HOR performance. This work may not only provide a facile protocol for the controllable synthesis of HEA SNWs, but also promote the fundamental researches on HEAs for catalysis and beyond.

## Results

### Material synthesis and characterization

**Fig. 1:**
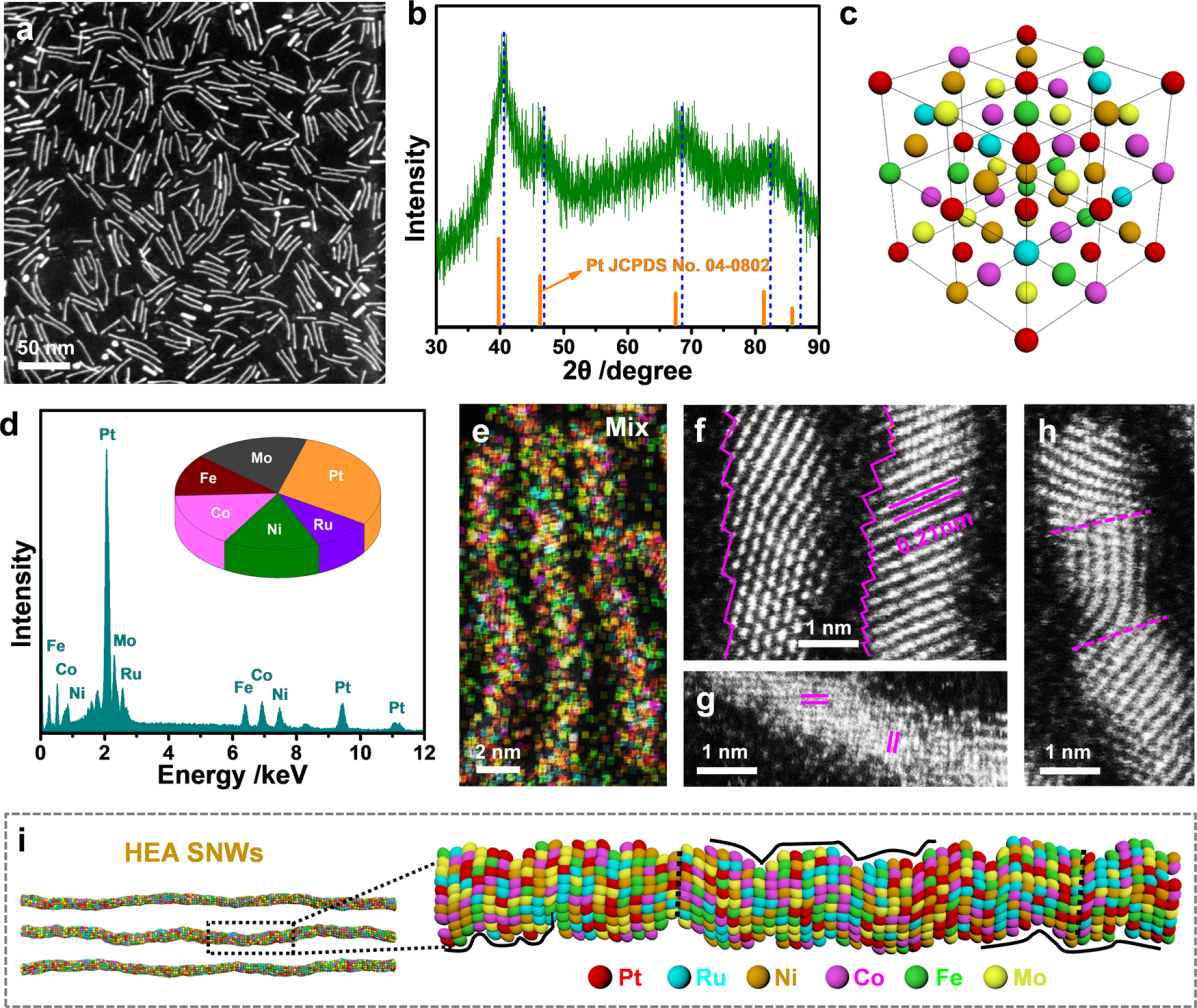
Characterizations of HEA SNWs. **a** HAADF-STEM image. **b** XRD pattern. **c** Crystal structure. **d** SEM-EDS and atomic ratio of different elements. **e** HAADF-STEM-EDS elemental mappings. **f**–**h** Aberration-corrected HRSTEM images. **i** 3D models and enlarged atomic model of HEA SNWs.

For the synthesis of HEA SNWs, we adopted platinum (II) acetylacetonate (Pt(acac)_2_), ruthenium (III) acetylacetonate (Ru(acac)_3_), nickel (II) acetylacetonate (Ni(acac)_2_), cobalt (III) acetylacetonate (Co(acac)_3_), iron (III) acetylacetonate (Fe(acac)_3_), and molybdenum hexacarbonyl (Mo(CO)_6_) as the metal precursors, oleylamine (OAm) as the solvent, stearyl trimethyl ammoium bromide (STAB) as the structure-directing agent, and glucose as the reducing agent, respectively. Transmission electron microscopy (TEM) and high-angle annular dark-field scanning TEM (HAADF-STEM) images show that the obtained HEA SNWs has a mean diameter of 1.8 ± 0.3 nm (Fig. 1a and Supplementary Fig. 1). The characteristic peaks in the X-ray diffraction (XRD) pattern are ascribed to face-centered cubic (*fcc*) structure of Pt (JCPDS No. 04−0802), and the positive shifts of peaks in XRD pattern imply the formation of alloy (Fig. 1b). On the other hand, the weakening and broadening of peaks in the XRD pattern could be attributed to the lattice distortion in HEA SNWs^18,19^. A scheme was thus provided to better understand the structure of HEA SNWs (Fig. 1c). Scanning electron microscope energy-dispersive X-ray spectroscopy (SEM-EDS) shows that the composition of HEA SNWs is Pt/Ru/Ni/Co/Fe/Mo = 29.6/9.3/15.2/15.6/12.2/18.1 (Fig. 1d), which is close to that from inductively coupled plasma optical emission spectroscopy (ICP-OES) measurement (Pt/Ru/Ni/Co/Fe/Mo = 28.5/6.8/18.6/15.2/10.7/20.2 (Supplementary Fig. 2). Moreover, both HAADF-STEM-EDS elemental mappings and line scan analysis show that all the metals are uniformly distributed in the HEA SNWs (Fig. 1e and Supplementary Fig. 3). Furthermore, the aberration-corrected high-resolution STEM (HRSTEM) images show that the obtained HEA SNW has a distorted structure with abundant atomic steps and defect-rich lattice mismatch (Fig. 1f−h). In addition, a 3D atomic model was provided to depict the structure of HEA SNWs (Fig. 1i), which has vividly revealed the unique structure of HEA SNWs with plentiful surface atomic steps and numerous facet boundaries.

### HOR performance of PtRuNiCoFeMo HEA SNWs

**Fig. 2:**
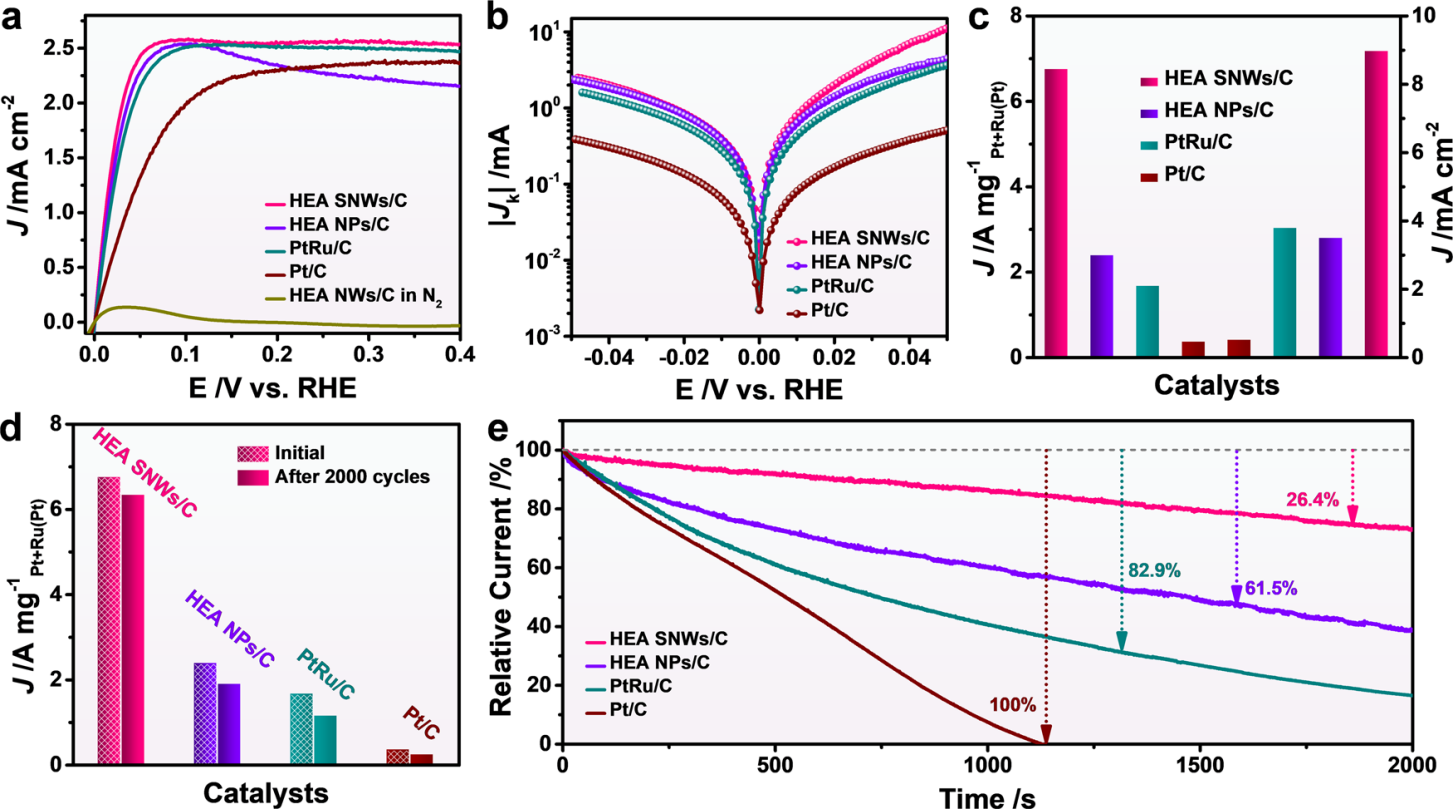
HOR performance evaluation of HEA SNWs/C and other catalysts. **a** Polarization curves in H_2_-saturated 0.1 M KOH. **b** Tafel plots. **c** Normalized mass activity and specific activity at an overpotential of 50 mV vs. RHE. **d** Normalized mass activity and specific activity before and after 2000 cycles of ADTs. **e** Relative current-time chronoamperometry response of different catalysts in 1000 ppm CO/H_2_-saturated 0.1 M KOH at 100 mV vs. RHE.

To evaluate the potential applications of the as-prepared HEAs, PtRuNiCoFeMo HEA SNWs were used as catalyst for HOR, a significant process for hydrogen fuel cell. Prior to HOR test, HEA SNWs were loaded on Vulcan XC-72R carbon (HEA SNWs/C, Supplementary Fig. 10a), and home-made HEA NPs (Supplementary Fig. 9) loaded on Vulcan XC-72R C (HEA NPs/C), commercial PtRu/C and Pt/C were selected as the references (Supplementary Fig. 10b−d and Fig. 11). Fourier transform-infrared (FT-IR) spectrum of HEA SNWs shows that most of the ligands have been removed from the surface of HEA SNWs/C (Supplementary Fig. 12). From the cyclic voltammograms (CVs) curves of various catalysts obtained in 0.1 M HClO_4_ solution at a scan rate of 50 mV s^−1^ (Supplementary Fig. 13), we can see that the presence of Ru significantly broadens the electrical double-layer capacitor^20,21^. It is found that the anode current of HEA SNWs/C increases sharply with the potential (Fig. 2a). In particular, HEA SNWs/C exhibits a high and steady current density with the increased potential, whereas a gradual decay in the current density was observed for HEA NPs/C, suggesting that HEA SNWs/C can be serve as a highly efficient catalyst for HOR^22^. Moreover, the HEA SNWs/C for HOR was evaluated in N_2_-saturated 0.1 M KOH solution to measure the anodic current. As shown in Fig. 2a (yellow line), the current density is ~0 when the potential is over 0.1 V, and the weak current density at 0–0.1 V is ascribed to Ru oxidation, which confirms the occurrence of HOR in H_2_-saturated 0.1 M KOH solution. The Koutecky–Levich plot was then collected to verify the electron transport process (Supplementary Fig. 14). It is found that the slopes for HEA NPs/C, PtRu/C, and Pt/C are 10.78, 11.36, 16.95 mA cm_disk_^−2^ rpm^1/2^, respectively. In contrast, the slope for HEA SNWs/C is 14.06 mA cm_disk_^−2^ rpm^1/2^, which is close to the theoretical value (15.06 mA cm_disk_^−2^ rpm^1/2^), further confirming the H_2_ mass-transport controlled process (Supplementary Fig. 15)^23,24^. Figure 2b shows the kinetic currents (*J*_k_) as a logarithmic function vs. the potential for HEA SNWs/C, HEA NPs/C, PtRu/C, and Pt/C. The value of exchange current (*I*_0_) of HEA NWs/C is 1.26 mA, which is 1.5, 2.4, and 4.5 times to that of HEA NPs/C (0.84 mA), PtRu/C (0.52 mA), and Pt/C (0.28 mA), respectively. To quantitatively compare the HOR activity of each catalyst, we calculated the mass activity and specific activity normalized with the loading amount of noble metal and electrochemical surface area (ECSA), respectively. The ECSAs of different catalysts were determined by the CO stripping curves (Supplementary Fig. 16 and Supplementary Table 1). As shown in Fig. 2c and Supplementary Table 2, the mass activity of HEA SNWs/C for HOR reaches 6.75 A mg_Pt+Ru_^−1^ at 50 mV vs. reversible hydrogen electrode (RHE), which is 2.8, 4.1, and 19.8 times higher than that of HEA NPs/C (2.37 A mg_Pt+Ru_^−1^), PtRu/C (1.65 A mg_Pt+Ru_^−1^) and Pt/C (0.34 A mg_Pt+Ru_^−1^), respectively. On the other hand, the specific activities of HEA NWs/C, HEA NPs/C, PtRu/C, and Pt/C are 8.96, 3.47, 3.77, and 0.48 mA cm^−2^, respectively, indicating the superior HOR activity of HEA SNWs/C to the references (Supplementary Table 2). Moreover, the stability was evaluated by measuring the HOR activities of different catalysts after 2000 cycles of ADTs (Supplementary Fig. 17). Only 6.2% loss in mass activity was observed after 2000 cycles of ADT for HEA SNWs/C. In sharp contrast, the HOR activities decrease by 20.6%, 31.5%, and 35.3% for HEA NPs/C, PtRu/C, and Pt/C, respectively, indicating the superior stability of HEA SNWs/C to commercial PtRu/C and Pt/C (Fig. 2d). Moreover, CO stripping experiments were performed on HEA SNWs/C and Pt/C after 2000 cycles of ADT to further study the stability. It is found that the ECSA of HEA SNWs/C decreases by 22.3% after 2000 cycles, which is lower than that of Pt/C (30.6%), suggesting the enhanced stability of HEA SNWs/C (Supplementary Fig. 18). Additionally, the morphologies and compositions of the spent catalysts were investigated. Compared to the spent PtRu/C and Pt/C catalysts, which aggregate after ADT (Supplementary Fig. 19), the morphology and composition of the spent HEA SNWs/C are maintained, indicating that HEA SNWs can serve as the stable catalyst for HOR (Supplementary Fig. 20). Considering the resistance to CO poisoning of catalyst is a key factor for hydrogen fuel cell^25–27^, the HOR performance of HEA SNWs/C and other references were investigated in the presence of 1000 ppm CO. It is noted that the limiting current density of HEA SNWs/C decreases only by 5.4% at 100 mV vs. RHE, which is smaller than those of HEA NPs/C (6.2%), PtRu/C (21.3%) and Pt/C (22.7%), suggesting the significantly enhanced resistance to CO poisoning of HEA SNWs/C (Supplementary Fig. 21). Moreover, the stability of catalyst was further evaluated using the chronoamperometry method at 100 mV vs. RHE in the presence of 1000 ppm CO. The HOR activity decreases by 26.4% after 2000 s (Fig. 2e). In contrast, the HOR activities of HEA NPs/C and PtRu/C decrease by 61.5% and 82.9%, respectively, and the commercial Pt/C completely deactivates after 1130 s. The aforementioned results imply that HEA SNWs/C can serve as a highly active and stable catalyst for alkaline HOR (Supplementary Table 3).

### Mechanism investigation

**Fig. 3:**
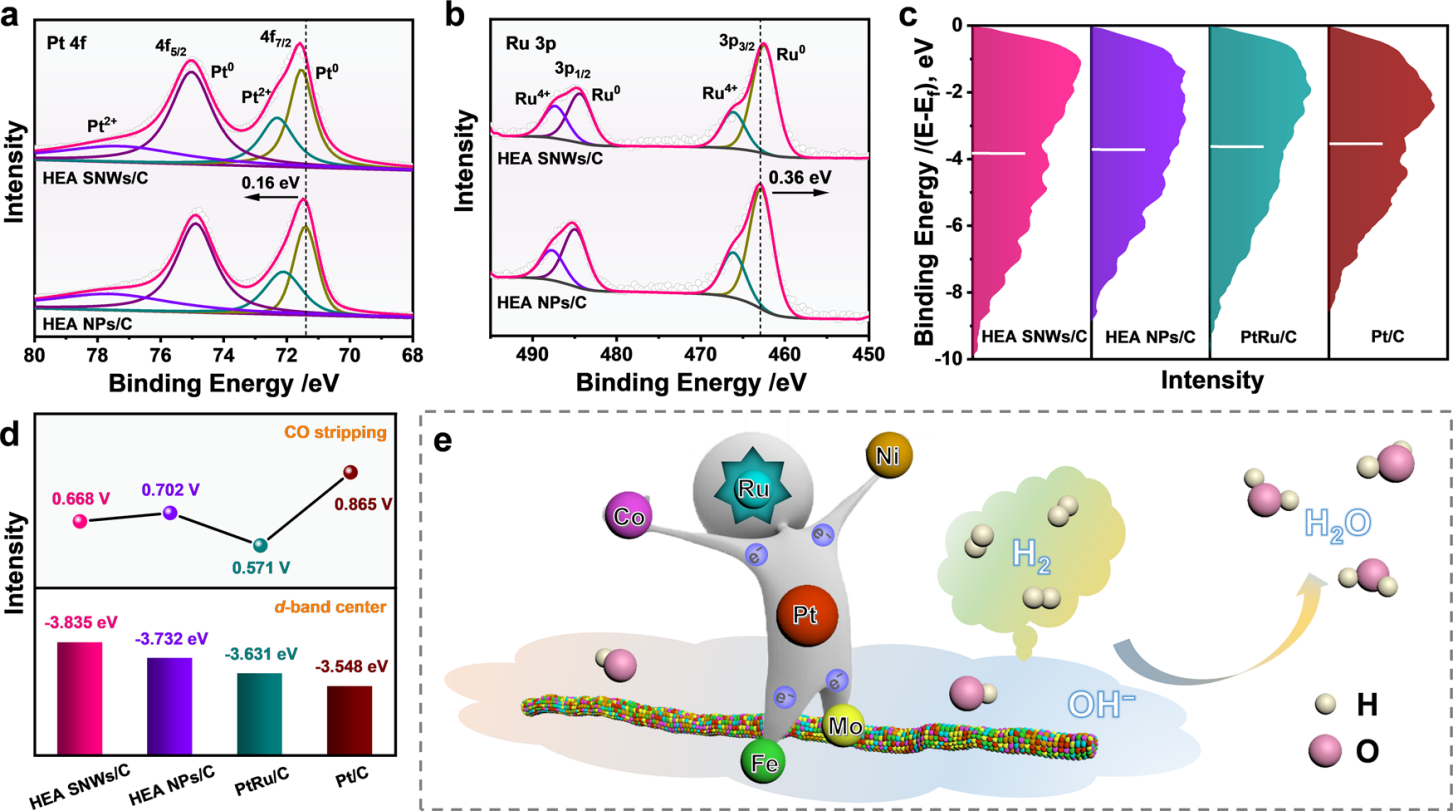
Surface valence band photoemission spectra analysis. **a** Pt 4*f* XPS spectra. **b** Ru 3*p* XPS spectra. **c** *d*-band center of different catalysts. The vertical lines indicate the *d*-band centers of samples relative to the Fermi level. **d** CO stripping potentials and *d*-band center positions of different catalysts. **e** Schematic illustration of HOR over HEA SNWs/C.

To reveal the mechanism for the enhanced HOR performance over HEA SNWs/C, the surface properties of catalyst was studied by X-ray photoelectron spectroscopy (XPS) measurement. It is noted that all the metal elements in HEA SNWs are mixed with metal state and oxidation state. Compared to HEA NPs/C, the peak in the Pt^0^ 4 *f* XPS spectrum of HEA SNWs/C positively shifts by 0.13 eV (Fig. 3a), while the peak in the Ru^0^ 3*p* XPS spectrum negatively shifts by 0.36 eV (Fig. 3b), indicating that electrons might transfer from Pt to Ru in HEA SNWs. On the other hand, the Ni^0^ 2*p*, Co^0^ 2*p*, Fe^0^ 2*p* and Mo^4+^ 3*d* XPS spectra of HEA SNWs positively shift by 0.26, 0.17, 0.93 and 0.25 eV, respectively, in comparison to HEA NPs (Supplementary Fig. 22). Considering that only Ru^0^ 3*p* XPS spectrum negatively shifts by 0.36 eV, we might conclude that the positive shifts of Ni^0^ 2*p*, Co^0^ 2*p*, Fe^0^ 2*p*, and Mo^4+^ 3*d* XPS spectra were attributed to the electron transfer from Ni, Co, Fe and Mo to Ru in HEA SNWs. To further study the influence of electron transfer on the binding strength of reactants and intermediates during HOR, we compared the *d*-band center of various catalysts. Compared to values of HEA NPs/C (−3.732 eV), PtRu/C (−3.631 eV) and Pt/C (−3.548 eV), the *d*-band center of HEA SNWs/C shifts upwards to −3.835 eV (Fig. 3c, d), which thereby weakens the adsorption of H but facilitate the adsorption of OH intermediate^28,29^. Considering the significant effect of Ru on HOR that can facilitate the adsorption of OH^*^ species^30,31^, we evaluated the HOR performance over HEA SNWs and PtNiCoFeMo HEA SNWs/C. The mass activity of HEA SNWs/C is 11.8 times higher than that of PtNiCoFeMo HEA SNWs/C (Supplementary Fig. 23a, b). Moreover, compared to the PtNiCoFeMo HEA SNWs/C, HEA SNWs/C displays significantly improved resistance to CO poisoning (Supplementary Fig. 23c). In particular, the HOR activity in the presence of 1000 ppm CO decreases by 26.4% after 2000 s when HEA SNWs/C was used as catalyst, which is much smaller than that of PtNiCoFeMo HEA SNWs/C (85.2%), confirming the significance of Ru on HOR. Additionally, the position of the CO stripping peak of HEA SNWs/C shifts positively compared with Pt/C, which further suggests that the presence of Ru significantly improves the resistance to CO poisoning (Fig. 3d and Supplementary Fig. 16). The aforementioned results imply that the strong electronic interaction between different elements in HEA SNWs can regulate the adsorption abilities of H and OH species, and then enhances the alkaline HOR activity (Fig. 3e).

**Fig. 4:**
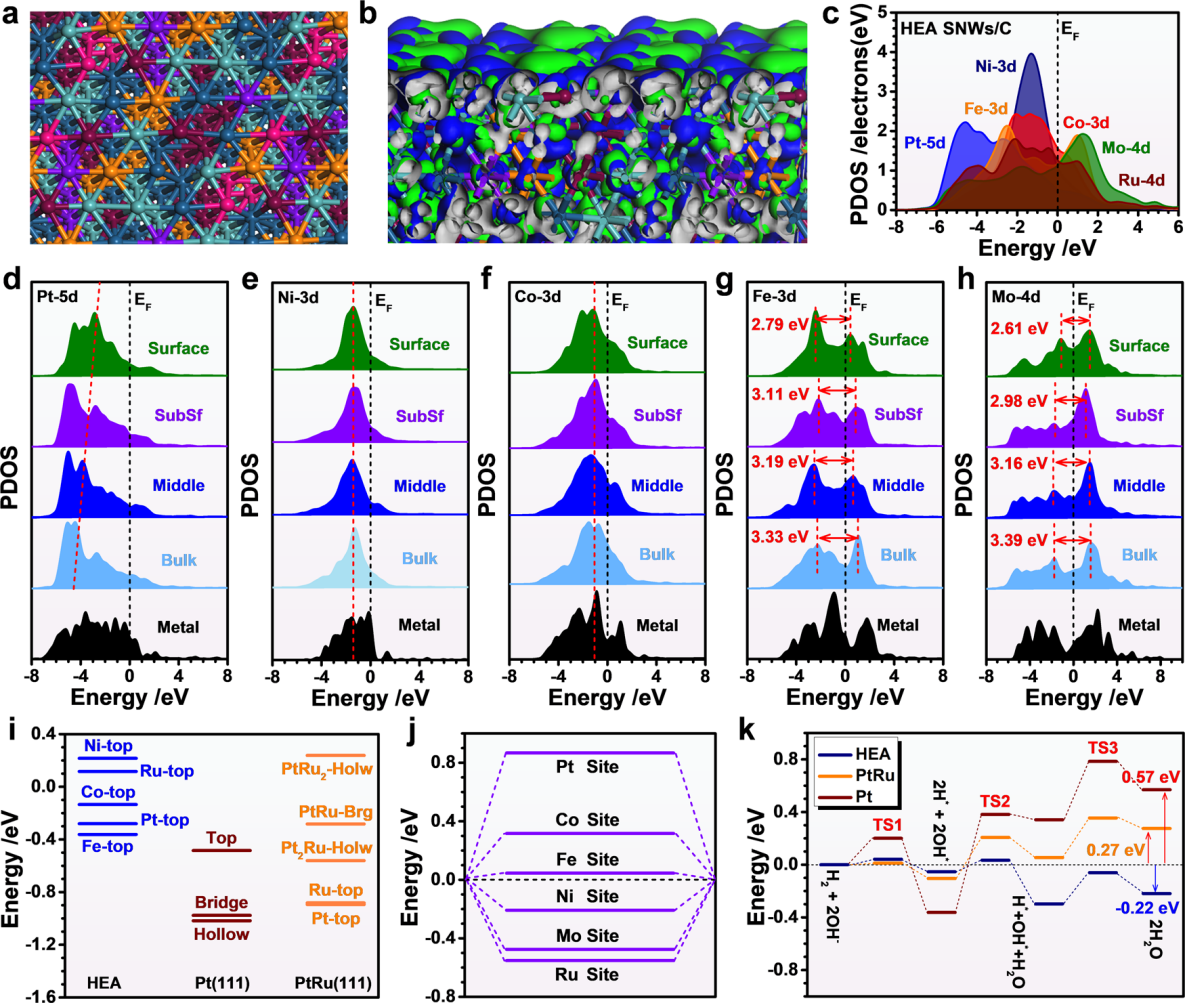
DFT calculations of HOR paths on HEA SNWs. **a** The top view after geometry optimizations. The dark green, brown, purple, orange, light green, and pink balls represent Pt, Ni, Co, Fe, Mo, and Ru atoms, respectively. **b** The 3D contour plot of electronic distributions near the Fermi level. **c** The PDOSs. Site-dependent PDOSs of **d** Pt-5*d*, **e** Ni-3*d*, **f** Co-3*d*, **g** Fe-3*d* and **h** Mo-4*d*. **i** The HBE comparison between HEA SNWs/C, Pt (111) and PtRu (111). Holw = Hollow sites. Brg = Bridge sites. **j** The OH binding energy on HEA SNWs/C. **k** The energetic trend of HOR on HEA SNWs/C, Pt (111) and PtRu (111).

DFT calculations were conducted to further investigate the mechanism for the enhanced HOR performance. It is noted that both Ni and Ru sites show subtle distortions while the overall HEA SNWs remain stable, which confirms the stability of HEA SNWs/C (Fig. 4a, b). With respect to the electronic structures, the strong coupling between the bonding and anti-bonding orbitals on the HEA surfaces delivers a high HOR activity with efficient site-to-site electron transfer between different metal sites. Moreover, the detailed electronic structures of HEA SNWs are also demonstrated by the projected partial density of states (PDOSs) (Fig. 4c)^32^. Notably, the evident overlaps between *d*-orbitals are observed, indicating the strong bonding between different metals. In particular, we notice the evident sharp Ni 3*d* orbitals locate near −1.30 eV, which is a key indicator for the strong adsorptions of H^*^ and OH^*^. Meanwhile, Co 3*d* orbitals show a similar position with the Ni 3*d* orbitals, which may further facilitate the electron transfer. Similar phenomena were observed for Fe 3*d* and Mo 4*d* orbitals, and the overlap of *d* orbital might not only enhance the electron transfer, but also impose the pinning effect on the 3*d* orbitals of Ni and Co sites for a robust electroactivity. On the other hand, Pt 5*d* orbital locates at the furthest position to Fermi level, suggesting that Ru plays as the electron reservoir to balance the valence state of HEA NWs during electrocatalysis. Moreover, the Ru 4*d* ranges from −6.0 to +4.0 eV, which enables the flexible electronic modulations and accelerates the site-to-site electron transfer on the HEA surfaces. Furthermore, the site-dependent electronic structure of each element in HEA SNWs has been demonstrated. As shown in Fig. 4d, the Pt-5*d* center gradually approaches to the Fermi level from the bulk (cyan color) to the surface of HEA SNWs (green color), indicating that the H_2_ adsorption becomes much stronger on the surface than on the bulk of HEA SNWs. It is noted that the overall Pt-5*d* orbital is further away from the Fermi level compared to the pure Pt metal (black color), indicating that HEA SNWs has an appropriate level of Pt-5*d* band for H_2_ adsorption and therefore exhibits enhanced HOR activity. The Ni-3*d* orbital of pure Ni metal (black color) is much broader than those of HEA SNWs (Fig. 4e), suggesting that the localized electron density of Ni-3*d* orbital in HEA SNWs is much higher than that of pure Ni metal, which can bring more possibilities in charge transfer and bonding chances with OH^*^. Besides, from the bulk to the surface, the gradual absence of the e_g_-t_2g_ band splitting of Co 3*d* orbitals leads to the efficient intra-orbital e_g_-t_2g_ electron transfer rather than transfer between *d*-*d* orbitals, which further promotes the site-to-site electron transfer from catalysts to adsorbates (Fig. 4f). It is also noted that the electronic structure of Co in HEA SNWs are similar from the bulk to the surface. Such a robust electronic structure is attributed to the double pinning effect by the shielding effect from neighboring Fe/Mo and Ru orbitals. For Fe sites, the e_g_-t_2g_ band splitting for 3*d* orbitals decreases from 3.33 to 2.79 eV from the bulk site to the surface sites, supporting a more efficient electron transfer towards OH^*^ (Fig. 4g). Similar decreases on the e_g_-t_2g_ band splitting were observed for Mo-4*d* orbitals, and the energy barrier significantly reduces from 3.39 to 2.61 eV from the bulk to the surface (Fig. 4h). The HEA SNWs has relaxed the forbidden rule of intra-orbital e_g_-t_2g_ electron transfer, which annihilates the gap between the e_g_-t_2g_ splitting of the Fe-3*d* bands and Mo-4*d* bands. The smaller gap between the band further lowers the barrier for the site-to-site electron transfer on the surface. The evolutions of electronic structures in Fe and Mo sites show a similar trend towards the decrease of the e_g_-t_2g_ splitting. This not only improves the site-to-site electron transfer on the surface, but also strengthens the protection effect of valence states for Ni and Co sites. In addition, we have performed the further detailed characterizations of electronic structures and confirmed the improved electroactivity of HEA surface, which guarantees the efficient HOR (Supplementary Fig. 24).

In addition, we compared the hydrogen binding energy (HBE) and the oxhydryl binding energy (OHBE) of HEA SNWs/C^33–35^. Compared with the strong bindings of H^*^ on Pt (111) and PtRu (111), the binding strength of H^*^ on HEA SNWs/C has been significantly weakened to an appropriate level near the 0 eV (an ideal value for H^*^ binding), leading to the improved HOR activity over HEA SNWs/C (Fig. 4i). For OHBE, despite the OH^*^ binding strength display a trend of Pt < Co < Fe < Ni  < Mo < Ru, the binding energy of OH^*^ on Ru is nearly −0.6 eV, suggesting that the moderate adsorption of OH^*^ on HEA SNWs/C (Fig. 4j). It has been well known that the adsorbed OH^*^ plays a vital role to remove the adsorbed proton in the form of H_2_O, and therefore significantly enhances the alkaline HOR activity (Fig. 4j and Supplementary Fig. 25)^36–38^. On the other hand, the overall reaction on HEA is exothermic with an energy release of 0.22 eV, indicating the rapid desorption of H_2_O. In sharp contrast, the overall reactions on Pt and PtRu are endothermic with energies of 0.57 and 0.27 eV, respectively (Fig. 4k). Note that the energy barrier for H_2_O formation on HEA surface is as low as 0.08 eV, which is much lower than that of Pt and PtRu, further confirming the superior HOR activity of HEA to Pt and PtRu (Fig. 4k).

## Discussion

In summary, we have demonstrated a successful synthesis of PtRuNiCoFeMo HEA SNWs with abundant surface atomic steps and facet boundaries for efficient HOR. Compared with the state-of-the-art PtRu/C and Pt/C catalysts, HEA SNWs/C exhibits advantages, including high activity, enhanced stability, and promising resistance to CO poisoning, being a promising catalyst for alkaline HOR. In particular, the mass activity and specific activity of HEA SNWs reach 6.75 A mg_Pt+Ru_^−1^ and 8.96 mA cm^−2^, which are 2.8/2.6, 4.1/2.4, and 19.8/18.7 times higher than those of HEA NP/C, commercial PtRu/C and Pt/C, respectively. DFT calculations reveal that the strong synergy significantly varies the electronic properties of different elements in HEA SNWs, which further regulates the HBE and OHBE. This work not only provides a viable synthetic route for the fabrication of Pt-based HEA subnano/nano materials, but also promotes the fundamental researches on catalysis and beyond.

## Methods

### Chemicals

Pt(acac)_2_ (97%), Ni(acac)_2_ (95%), Co(acac)_3_ (98%), Fe(acac)_3_ (98%), and STAB (98%) were purchased from Sigma-Aldrich. Ru(acac)_3_ (97%) was supplied by Alfa Aesar. Mo(CO)_6_ (98%) was purchased from Strem Chemicals Inc. Glucose (C_6_H_12_O_6_, analytical reagent), potassium hydrate (KOH, analytical reagent), ethanol (C_2_H_6_O, analytical reagent, ≥99.7%), cyclohexane (C_6_H_12_, analytical reagent, ≥99.7%) and isopropanol (C_3_H_8_O, analytical reagent, ≥99.7%) were purchased from Sinopharm Chemical Reagent Co. Ltd. OAm was supplied by Aladdin. HClO_4_ (analytical reagent, 70–72%) was purchased from Tianjin Zhengcheng Chemical Products Co. Ltd. All chemical reagents were used as received without further purification. All aqueous solutions were prepared using deionized water with a resistivity of 18.2 MΩ cm^−1^.

### Materials synthesis

In a typical synthesis of PtRuNiCoFeMo HEA SNWs, 9.8 mg Pt(acac)_2_, 5.0 mg Ru(acac)_3_, 3.8 mg Ni(acac)_2_, 7.0 mg Co(acac)_3_, 7.0 mg Fe(acac)_3_, 10 mg Mo(CO)_6_, 60 mg glucose, and 39.3 mg STAB were dissolved in 5 mL OAm, followed by ultrasonication for 2 h. The mixture was then heated in an oil bath at 210 °C for 5 h. The cooled product was collected by centrifugation and washed three times with a cyclohexane/ethanol (v/v = 1:9) mixture. For the synthesis of PtRuNiCoFeMo HEA NPs, all of the conditions are similar to those of HEA SNWs except for the additions of STAB and glucose.

### Characterizations

XRD measurement was conducted on a SmartLab-SE powder diffractometer equipped with a Cu radiation source (*λ* = 0.15406 nm). SEM-EDS was observed through ZEISS Sigma 300 field emission scanning electron microscope. TEM was operated on JEM-1400 TEM at an accelerating voltage of 100 kV. HRTEM was conducted on a FEI Tecnai F30 TEM at an accelerating voltage of 300 kV. HAADF-STEM-EDS was conducted on a FEI Titan Cubed Themis G2300. The X-ray photoelectron spectroscopy spectra were collected by XPS (Thermo Scientific, ESCALAB 250 XI). The carbon peak at 284.6 eV was used as the reference to correct for charging effects. The concentrations of the catalysts were determined by ICP-OES (ICAP 7000, ThermoFisher, USA). FT-IR was performed with KBr in the range of 4000–400 cm^−1^ (Nicolet iS50).

### Electrochemical measurements

The electrocatalytic properties toward HOR were evaluated using a RDE (Pine, diameter of 5 mm) with CHI 760E Electrochemical Workstation. A SCE and a graphite rod were used as the reference electrode and counter electrode, respectively. HOR measurements were performed in 0.1 M KOH solutions purged with high-purity H_2_. The sweep rate was 5 mV s^−1^ to collected the HOR polarization curves. The accelerated durability tests were carried out in 0.1 M KOH between −0.1 V and 0.4 V vs. RHE at a scan rate of 500 mV s^−1^ for 2000 cycles. The CO antipoisoning tests were carried out in 0.1 M KOH purged with saturated 1000 ppm CO/H_2_, and the long-range stability test voltage was at 100 mV vs. RHE.

The kinetic current (*J*_k_) was calculated using the Koutecky–Levich equation.

1 $$\frac{1}{J}=\frac{1}{J_{{{{{\mathrm{k}}}}}}}+\frac{1}{J_{{{{{\mathrm{d}}}}}}}$$

where *J* is measured current and *J*_d_ is diffusion-limited current, which can be fitted to the Butler–Volmer equation and Levich equation:

2 $$J_{{{{{\mathrm{k}}}}}}={{J}}_{{{{{\mathrm{0}}}}}}\left({e}^{\frac{\alpha_{{{{{\mathrm{a}}}}}}{{{{{\rm{F}}}}}}{{{{{\rm{\eta }}}}}}}{{{{{{\rm{RT}}}}}}}}-{e}^{-\frac{\alpha_{{{{{\mathrm{c}}}}}}{{{{{\rm{F}}}}}}{{{{{\rm{\eta }}}}}}}{{{{{{\rm{RT}}}}}}}}\right)$$

3 $$J_{{{{{\mathrm{d}}}}}}=0{{{{{\rm{.62nF}}}}}}{D}^{3/2}{\nu }^{-1/6}C_0{\omega }^{1/2}={{BC}}_0{\omega }^{1/2}$$

in which *α*_a_ and *α*_c_ are the anodic and cathodic transfer coefficients (*α*_a_ + *α*_c_ = 1), *F* is the Faraday constant, *η* is the overpotential, *R* is the universal gas constant, *T* is the Kelvin temperature, *n* is the electrons number, *D* is the diffusion coefficient, *ν* is the viscosity coefficient, *C*_0_ is the solubility, *ω* is the rotating speed, and *B* is the Levich constant, respectively.

For the CO stripping measurement, CO gas was bubbled into 0.1 M HClO_4_ solution for 30 min. The electrode was quickly moved to a fresh 0.1 M HClO_4_ solution and recorded the two cycles to calculate CO stripping peak. The electrochemical surface area of the noble metals was obtained using Eqs. (4) and (5).

4 $${Q}_{{{{{{\rm{co}}}}}}-{{{{{\rm{adsorption}}}}}}}(C)=\frac{\int {{{{{\rm{i}}}}}}\,{{{{{\rm{dE}}}}}}({{{{{\rm{mA}}}}}}\,{{{{{\rm{V}}}}}})}{\nu ({{{{{\rm{mV}}}}}}/{{{{{\rm{s}}}}}})}$$

5 $${{{{{\rm{ECSA}}}}}}\left(\frac{{m}^{2}}{g}\right)=\left[\frac{{Q}_{{{{{{\rm{co}}}}}}-{{{{{\rm{adsorption}}}}}}}(C)}{420(\mu \frac{C}{{{{{{{\rm{cm}}}}}}}^{2}}){M}_{{{{{{\rm{Pt}}}}}}/({{{{{\rm{Pt}}}}}}+{{{{{\rm{Ru}}}}}})}({{{{{\rm{mg}}}}}})}\right]{10}^{5}$$

### Calculation setup

To investigate the electronic structures, we have utilized DFT calculations within CASTEP packages in this work^39^. To achieve accurate descriptions of the exchange-correlation energy, we applied the generalized gradient approximation (GGA) and Perdew–Burke–Ernzerhof (PBE) in all the calculations^40–42^. Meanwhile, we have set the cutoff energy of the plane-wave basis to be 380 eV based on the ultrafine quality. Meanwhile, we choose the ultrasoft pseudopotentials with the Broyden-Fletcher-Goldfarb-Shannon (BFGS) algorithm. For the k-point settings, we have applied the coarse quality for the energy minimizations^43^. Based on the experimental results, the HEA structure has been cleaved from the (111) surface of *fcc* Pt with five-layered thickness. We choose a 4 × 5 × 1 unit cell for the HEA structure with 100 atoms. The element ratios are consistent with the ICP-OES results of experiments, which show the component of 28.5/6.8/18.6/15.2/10.7/20.2 for Pt/Ru/Ni/Fe/Co/Mo. In this work, the slab model is constructed based on the random arrangements coding methods. We compare this slab model with other slab models based on slightly different atomic arrangements of the elements. After comparison, we select the model with the lowest formation energies as the most thermodynamically stable model in this work. Different binding positions are selected for HEA, Pt (111) and Pt (111) surface. For the HEA surfaces, we select the element-top sites for the proton adsorption. For Pt (111) surfaces, we select three different adsorption sites, including Pt-top, bridge and hollow sites. For the PtRu (111) surface, we select five different adsorption sites, including Pt-top, Ru-top, PtRu-bridge, Pt_2_Ru (Pt-Pt-Ru) hollow and PtRu_2_ (Pt-Ru-Ru) hollow sites. In PtRu (111) surface, PtRu_2_ hollow sites represent the hollow sites that constructed by one Pt and two Ru sites and the Pt_2_Ru hollow sites represent the hollow sites constructed by two Pt and one Ru sites. To guarantee relaxations, we introduce 20 Å vacuum space in the z-axis. For all the geometry optimizations, we have set the following criteria for the convergence: Hellmann-Feynman forces on the atom should not exceed 0.001 eV Å^−1^, the total energy difference should be less than 5 × 10^−5^ eV atom^−1^, and the inter-ionic displacement should not exceed 0.005 Å. For the reactions of HOR, the calculations of energetic trend are based on the following reactions (Eqs. 6–8)^36–38^.

6 $${{{{{\rm{Tafel}}}}}}\,{{{{{\rm{step}}}}}}:\,{{{{{{\rm{H}}}}}}}_{2}+2{{{{{{\rm{OH}}}}}}}^{-}\,=2{{{{{{\rm{H}}}}}}}^{\ast }+2{{{{{{\rm{OH}}}}}}}^{\ast }+2{{{{{{\rm{e}}}}}}}^{-}$$

7 $${{{{{\rm{Volmer}}}}}}\,{{{{{\rm{step}}}}}}:2{{{{{{\rm{H}}}}}}}^{\ast }+2{{{{{{\rm{OH}}}}}}}^{\ast }+2{{{{{{\rm{e}}}}}}}^{-}\,={{{{{{\rm{H}}}}}}}_{2}{{{{{\rm{O}}}}}}+{{{{{{\rm{H}}}}}}}^{\ast }+{{{{{{\rm{OH}}}}}}}^{\ast }+{{{{{{\rm{e}}}}}}}^{-}$$

8 $${{{{{{\rm{H}}}}}}}_{2}{{{{{\rm{O}}}}}}+{{{{{{\rm{H}}}}}}}^{\ast }+{{{{{{\rm{OH}}}}}}}^{\ast }+{{{{{{\rm{e}}}}}}}^{-}\,=2{{{{{{\rm{H}}}}}}}_{2}{{{{{\rm{O}}}}}}+2{{{{{{\rm{e}}}}}}}^{-}$$

## Supplementary information

Supporting Information

## Data Availability

The data that support the findings of this study are available from the corresponding author upon request. Source data are provided with this paper.

## Source data

Source data

## References

[1] Gasteiger HA, Marković NM (2009). Just a dream or future reality?. Science.

[2] Zhang H, Shen PK (2012). Advances in the high performance polymer electrolyte membranes for fuel cells. Chem. Soc. Rev..

[3] Cong YY, Yi BL, Song YJ (2018). Hydrogen oxidation reaction in alkaline media: from mechanism to recent electrocatalysts. Nano Energy.

[4] Long C (2019). Tuning the electronic structure of PtRu bimetallic nanoparticles for promoting the hydrogen oxidation reaction in alkaline media. Inorg. Chem. Front..

[5] Qin B (2018). A novel IrNi@PdIr/C core–shell electrocatalyst with enhanced activity and durability for the hydrogen oxidation reaction in alkaline anion exchange membrane fuel cells. Nanoscale.

[6] Strmcnik D (2013). Improving the hydrogen oxidation reaction rate by promotion of hydroxyl adsorption. Nat. Chem..

[7] Durst J (2014). New insights into the electrochemical hydrogen oxidation and evolution reaction mechanism. Energ. Environ. Sci..

[8] Long C (2019). Tuning the electronic structure of PtRu bimetallic nanoparticles for promoting the hydrogen oxidation reaction in alkaline media. Inorg. Chem. Front..

[9] Li Q (2019). The comparability of Pt to Pt-Ru in catalyzing the hydrogen oxidation reaction for alkaline polymer electrolyte fuel cells operated at 80 °C. Angew. Chem. Int. Ed..

[10] George EP, Raabe D, Ritchie RO (2019). High-entropy alloys. Nat. Rev. Mater..

[11] Tsai MH, Yeh JW (2014). High-entropy alloys: a critical review. Mater. Res. Lett..

[12] Wang X (2020). Continuous synthesis of hollow high-entropy nanoparticles for energy and catalysis applications. Adv. Mater..

[13] Shi P (2019). Enhanced strength-ductility synergy in ultrafine-grained eutectic high-entropy alloys by inheriting microstructural lamellae. Nat. Commun..

[14] Alfred L (2021). Maximize mixing in highly polyelemental solid solution alloy nanoparticles. Matter.

[15] Wu ZP (2021). Alloying-realloying enabled high durability for Pt-Pd-3d-transition metal nanoparticle fuel cell catalysts. Nat. Commun..

[16] Li H (2020). Fast site-to-site electron transfer of high-entropy alloy nanocatalyst driving redox electrocatalysis. Nat. Commun..

[17] Cui M (2021). High-entropy metal sulfide nanoparticles promise high-performance oxygen evolution reaction. Adv. Energy Mater..

[18] Xin Y (2020). High-entropy alloys as a platform for catalysis: progress, challenges, and opportunities. ACS Catal..

[19] Chang X, Zeng M, Liu K, Fu L (2020). Phase engineering of high-entropy alloys. Adv. Mater..

[20] Li J (2017). Experimental proof of the bifunctional mechanism for the hydrogen oxidation in alkaline media. Angew. Chem. Int. Ed..

[21] Zhang J (2021). Engineering the near-surface of PtRu3 nanoparticles to improve hydrogen oxidation activity in alkaline electrolyte. Small.

[22] Zhou Y (2020). Lattice-confined Ru clusters with high CO tolerance and activity for the hydrogen oxidation reaction. Nat. Catal..

[23] Sheng W, Gasteiger HA, Yang SHY (2010). Hydrogen oxidation and evolution reaction kinetics on platinum: acid vs alkaline electrolytes. J. Electrochem. Soc..

[24] Mao J (2020). Isolated Ni atoms dispersed on Ru nanosheets: high-performance electrocatalysts toward hydrogen oxidation reaction. Nano Lett..

[25] Lu SQ, Zhuang ZB (2017). Investigating the influences of the adsorbed species on catalytic activity for hydrogen oxidation reaction in alkaline electrolyte. J. Am. Chem. Soc..

[26] Wang LK (2019). Suppression of carbon monoxide poisoning in proton exchange membrane fuel cells via gold nanoparticle/titania ultrathin film heterogeneous catalysts. ACS Appl. Energy Mater..

[27] Sheng WC (2015). Correlating hydrogen oxidation and evolution activity on platinum at different pH with measured hydrogen binding energy. Nat. Commun..

[28] Pan Y (2019). Electronic structure and d-band center control engineering over M-doped CoP (M = Ni, Mn, Fe) hollow polyhedron frames for boosting hydrogen production. Nano Energy.

[29] Chen Z (2018). Tailoring the d-band centers enables Co4N nanosheets to be highly active for hydrogen evolution catalysis. Angew. Chem. Int. Ed..

[30] Wang Y (2015). Pt-Ru catalyzed hydrogen oxidation in alkaline media: oxophilic effect or electronic effect?. Energ. Environ. Sci..

[31] Takeguchi T (2012). Evidence of nonelectrochemical shift reaction on a co-tolerant high-entropy state Pt-Ru anode catalyst for reliable and efficient residential fuel cell systems. J. Am. Chem. Soc..

[32] Thomas AB (2019). High-Entropy Alloys as a Discovery Platform for Electrocatalysis. Joule.

[33] Qiu Y (2018). BCC-Phased PdCu alloy as a highly active electrocatalyst for hydrogen oxidation in alkaline electrolytes. J. Am. Chem. Soc..

[34] Wang G (2019). Unraveling the composition-activity relationship of Pt-Ru binary alloy for hydrogen oxidation reaction in alkaline media. J. Power Sources.

[35] Duan Y (2020). Bimetallic nickel-molybdenum/tungsten nanoalloys for high-efficiency hydrogen oxidation catalysis in alkaline electrolytes. Nat. Commun..

[36] Strmcnik D (2013). Improving the hydrogen oxidation reaction rate by promotion of hydroxyl adsorption. Nat. Chem..

[37] Alia SM, Pivovar BS, Yan Y (2013). Platinum-coated copper nanowires with high activity for hydrogen oxidation reaction in base. J. Am. Chem. Soc..

[38] Durst J (2014). New insights into the electrochemical hydrogen oxidation and evolution reaction mechanism. Energy Environ. Sci..

[39] Clark SJ (2005). First principles methods using castep. Z. Kristallogr.

[40] Hasnip PJ, Pickard CJ (2006). Electronic energy minimisation with ultrasoft pseudopotentials. Comput. Phys. Commun..

[41] Perdew JP, Burke K, Ernzerhof M (1996). Generalized gradient approximation made simple. Phys. Rev. Lett..

[42] Perdew JP (1992). Atoms, molecules, solids, and surfaces - applications of the generalized gradient approximation for exchange and correlation. Phys. Rev. B.

[43] Head JD, Zerner MC (1985). A broyden-fletcher-goldfarb-shanno optimization procedure for molecular geometries. Chem. Phys. Lett..

