# Publisher Correction: Subnanometer high-entropy alloy nanowires enable remarkable hydrogen oxidation catalysis

**DOI:** 10.1038/s41467-021-27395-1

**Published:** 2021-12-01

**Authors:** Changhong Zhan, Yong Xu, Lingzheng Bu, Huaze Zhu, Yonggang Feng, Tang Yang, Ying Zhang, Zhiqing Yang, Bolong Huang, Qi Shao, Xiaoqing Huang

**Affiliations:** 1 State Key Laboratory of Physical Chemistry of Solid Surfaces, College of Chemistry and Chemical Engineering, Xiamen University, Xiamen, 361005 China; 2 Guangzhou Key Laboratory of Low-Dimensional Materials and Energy Storage Devices, Collaborative Innovation Center of Advanced Energy Materials, School of Materials and Energy, Guangdong University of Technology, Guangzhou, 510006 China; 3 Shenyang National Laboratory for Materials Science, Institute of Metal Research, Chinese Academy of Sciences, Shenyang, 110016 China; 4 College of Chemistry, Chemical Engineering and Materials Science, Soochow University, Suzhou, 215123 China; 5 Department of Applied Biology and Chemical Technology, The Hong Kong Polytechnic University, Hung Hom, Kowloon, Hong Kong SAR China

**Keywords:** Electrocatalysis, Electrocatalysis, Nanowires

Correction to: *Nature Communications* 10.1038/s41467-021-26425-2, published online 29 October 2021.

The HTML version of this Article mislabelled the ‘Author guidance’ and ‘Source data’ files as their labels were inadvertently switched. This is now corrected and the Author guidance file has now been removed.

