# Supplementary material for: Subnanometer high-entropy alloy nanowires enable remarkable hydrogen oxidation catalysis
Source: Nat Commun. 2021 Oct 29;12:6261. doi: 10.1038/s41467-021-26425-2 (PMC8556242; doi:10.1038/s41467-021-26425-2)
Supplement: Supplementary file 1 — Supporting Information [file 41467_2021_26425_MOESM1_ESM.pdf]

Supporting Information

Subnanometer High-Entropy Alloy Nanowires Enable Remarkable Hydrogen Oxidation Catalysis

Zhan et al.

Supplementary Figures and Tables:

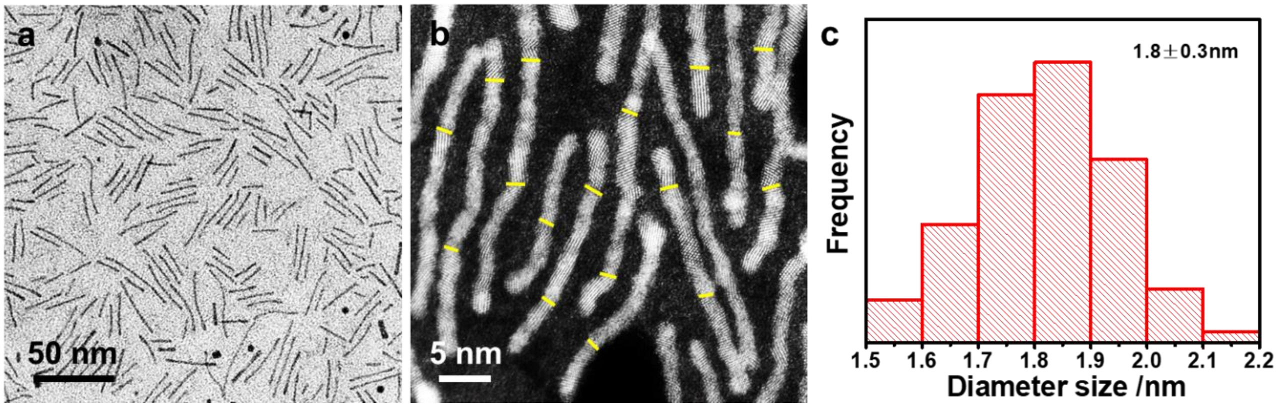

Supplementary Figure 1. (a) TEM image, (b) HAADF-STEM image, and (c) size distribution of PtRuNiCoFeMo HEA SNWs. The yellow dotted lines in (b) clearly reveal the presence of numerous facet boundaries in PtRuNiCoFeMo HEA SNWs.

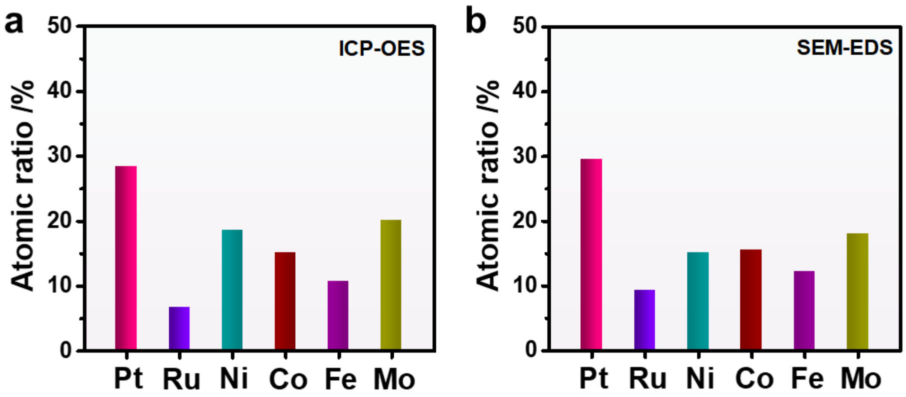

Supplementary Figure 2. The atomic ratios of different metals in PtRuNiCoFeMo HEA SNWs. Composition obtained from (a) ICP-OES and (b) SEM-EDS measurement.

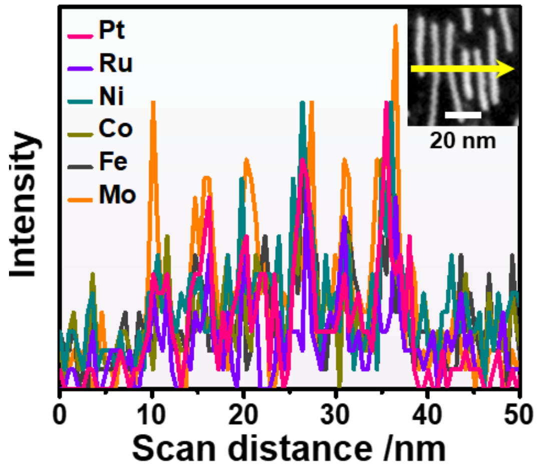

Supplementary Figure 3. Line scan analysis of PtRuNiCoFeMo HEA SNWs.

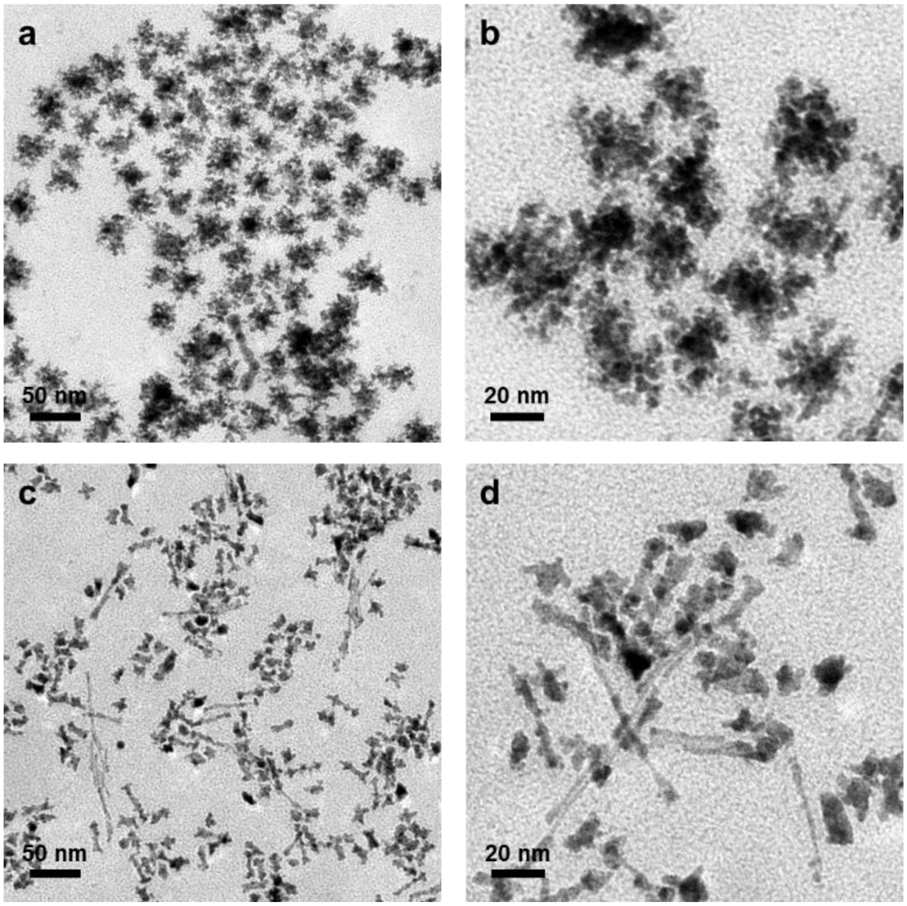

Supplementary Figure 4. TEM images of the products with the same reaction conditions as those of HEA SNWs except the use of (a, b) 8 mg $Pt(NH_3)_4(NO_3)_2$ and (c, d) 9.7 mg K_2PtCl_6.

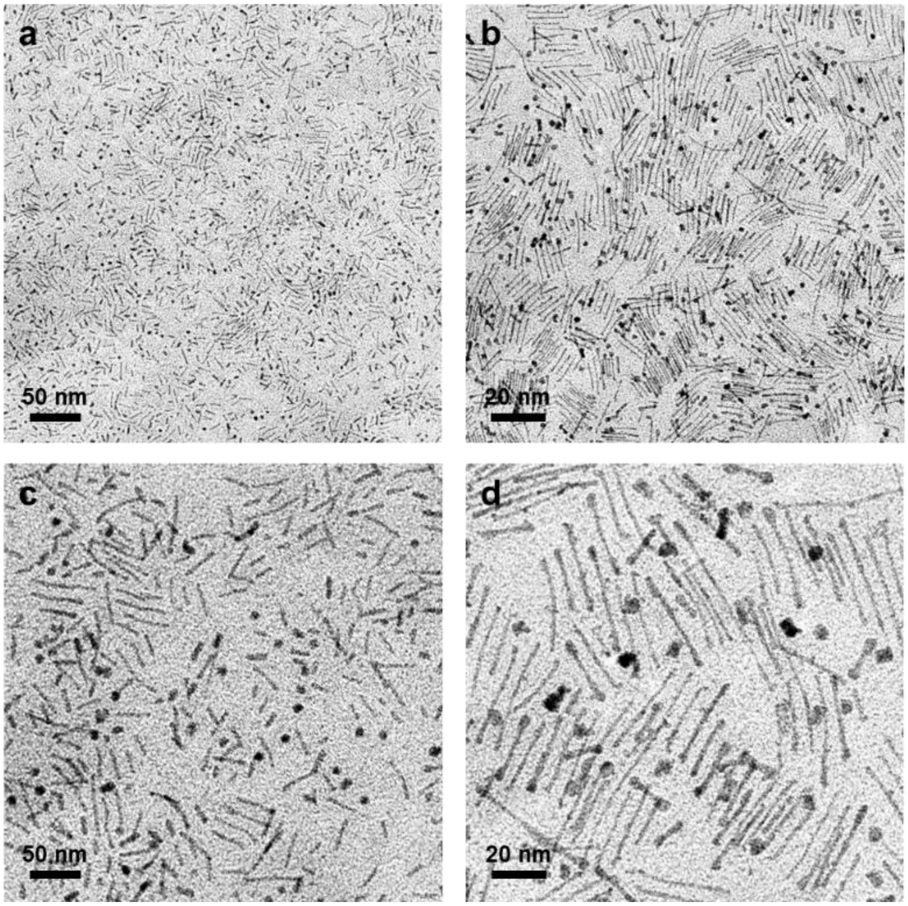

Supplementary Figure 5. TEM images of the products with the same reaction conditions as those of HEA SNWs except the use of (a, b) 2.7 mg $Ru_3(CO)_{12}$ and (c, d) 2.6 mg $RuCl_3 \cdot H_2O$.

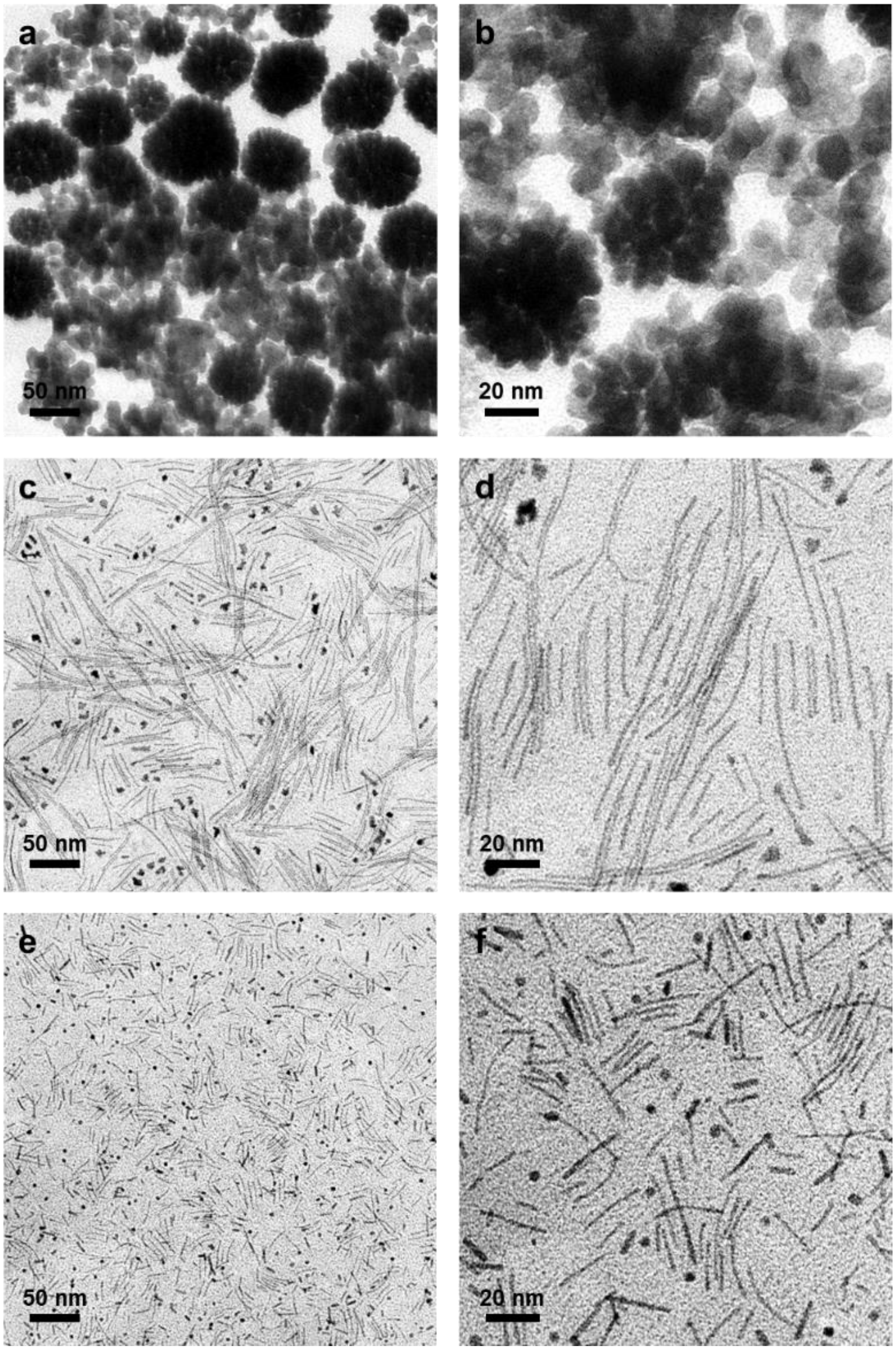

Supplementary Figure 6. TEM images of the products with the same reaction conditions as those of HEA SNWs except the use of (a, b) 0 mg $Mo(CO)_6$, (c, d) 5 mg $Mo(CO)_6$, and (e, f) 20 mg $Mo(CO)_6$.

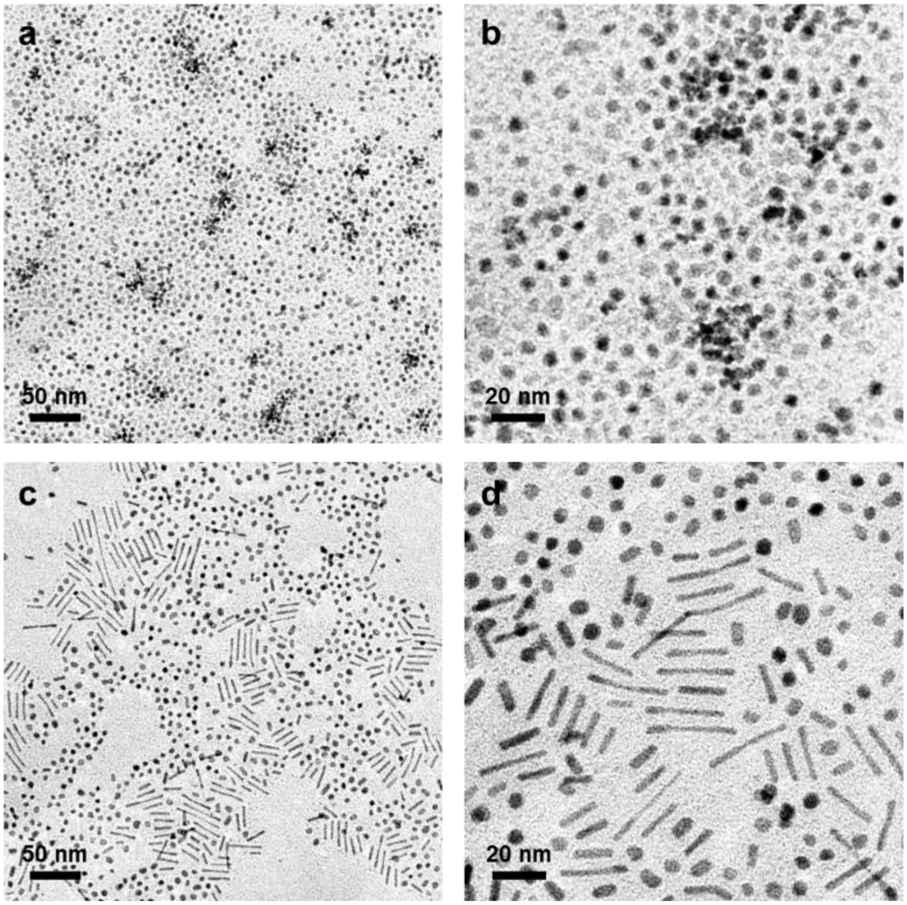

Supplementary Figure 7. TEM images of the products with the same reaction conditions as those of HEA SNWs except (a, b) without STAB and (c, d) replacing 60 mg glucose with 36 mg AA.

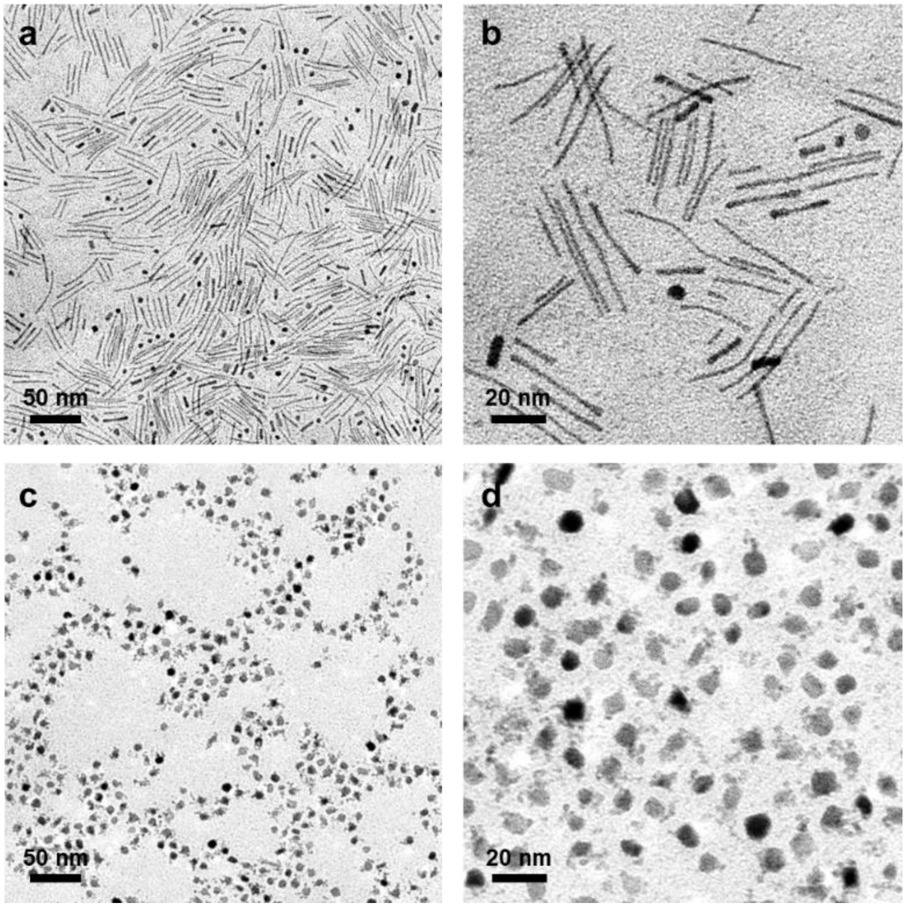

Supplementary Figure 8. TEM images of the products with the same reaction conditions as those of HEA SNWs except the use of (a, b) 4 mL OAm + 1 mL ODE and (c, d) 4 mL OAm + 1 mL OA.

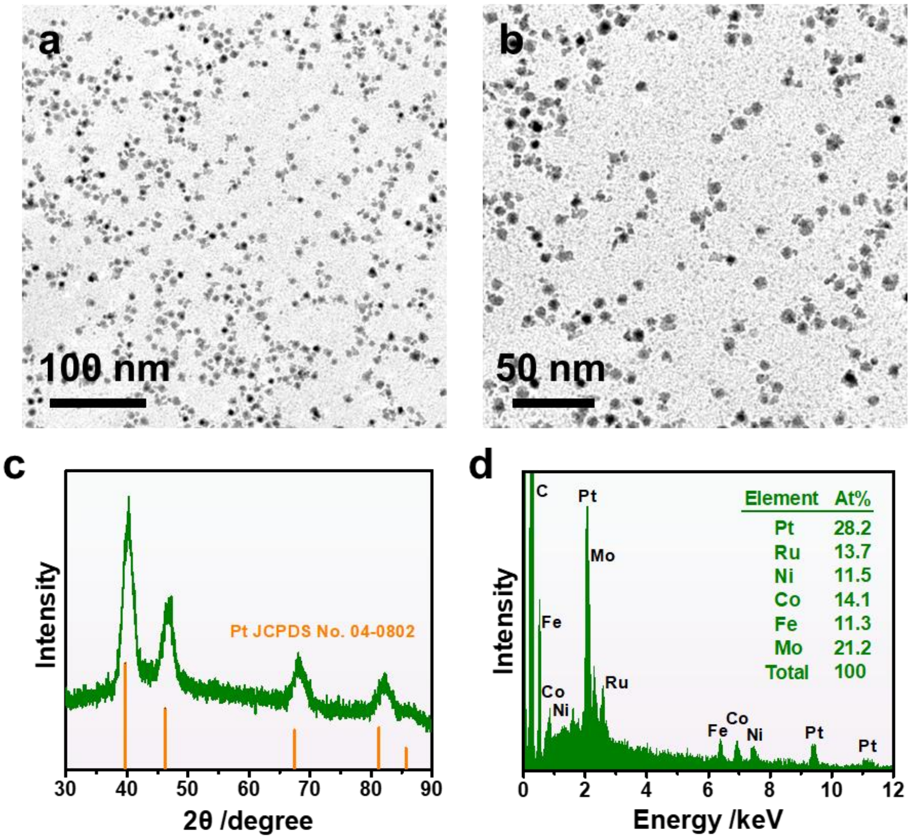

Supplementary Figure 9. (a, b) TEM images, (c) PXRD pattern, and (d) SEM-EDS of PtRuNiCoFeMo HEA NPs.

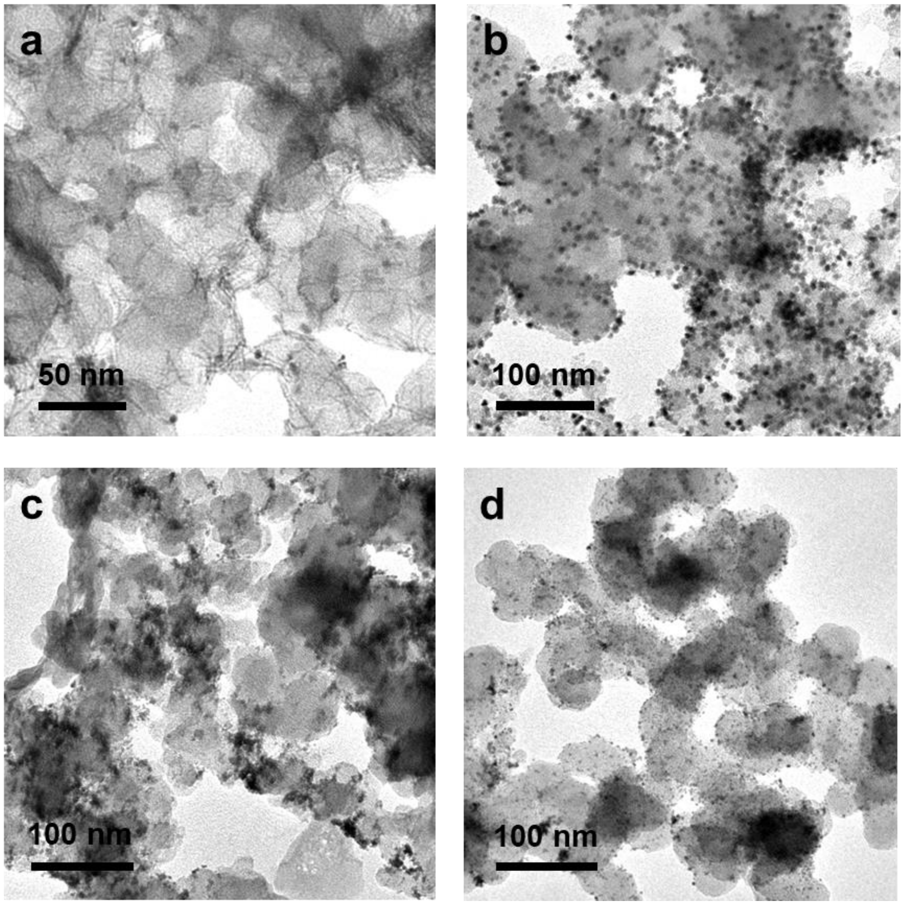

Supplementary Figure 10. TEM images of (a) HEA SNWs/C, (b) HEA NPs/C, (c) commercial PtRu/C, and (d) commercial Pt/C.

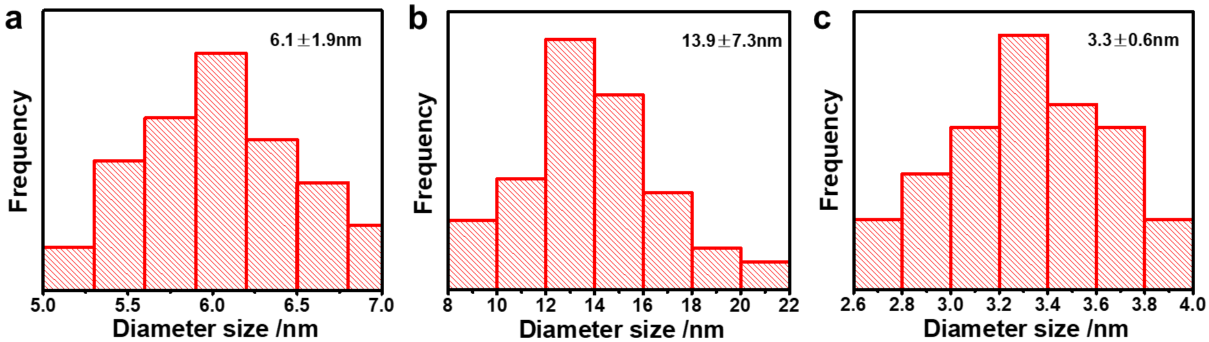

Supplementary Figure 11. Size distribution of (a) HEA NPs/C, (b) commercial PtRu/C, and (c) commercial Pt/C.

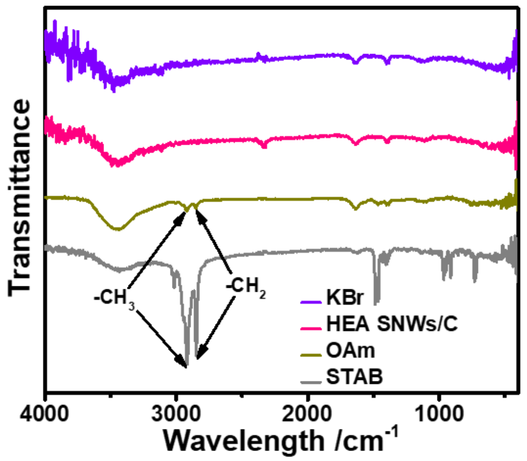

Supplementary Figure 12. FT-IR of KBr, HEA SNWs/C, OAm, and STAB.

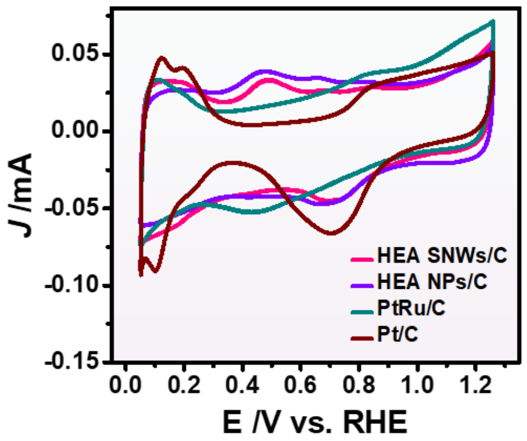

Supplementary Figure 13. CV curves of different catalysts in 0.1 M $HClO_4$.

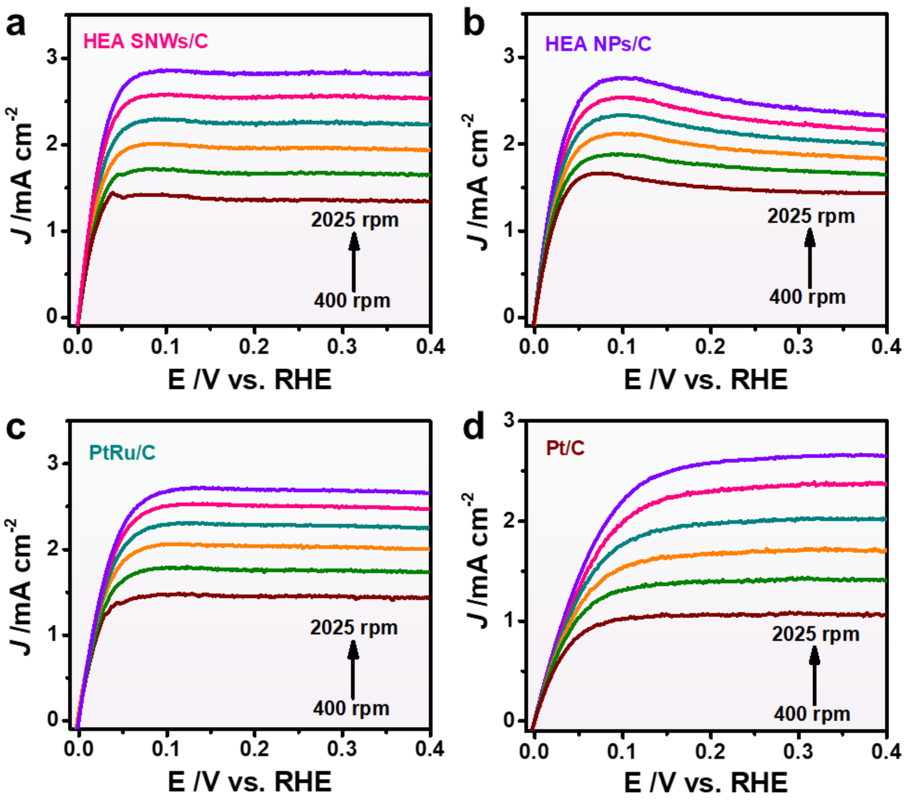

Supplementary Figure 14. Polarization curves of (a) HEA SNWs/C, (b) HEA NPs/C, (c) commercial PtRu/C, and (d) commercial Pt/C obtained at different rotating speeds (400, 625, 900, 1600, and 2025 rpm).

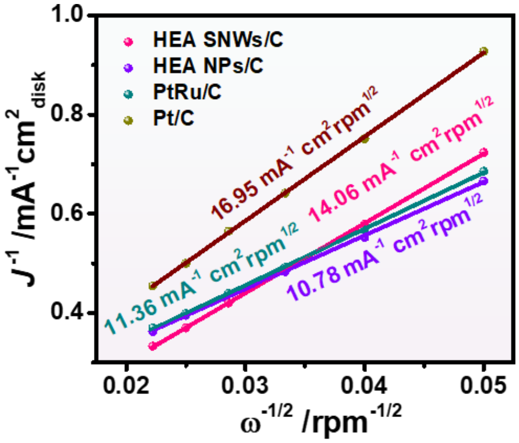

Supplementary Figure 15. Koutecky-Levich plots at an overpotential of 100 mV.

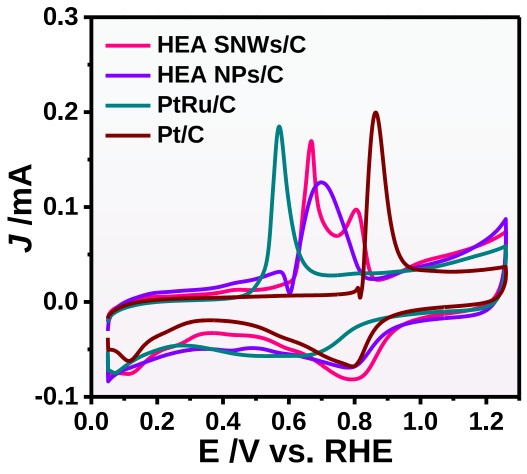

Supplementary Figure 16. CO-stripping curves of HEA SNWs/C, HEA NPs/C, commercial PtRu/C, and commercial Pt/C in 0.1 M $HClO_4$ solution at a scan rate of 20 mV s^{-1}.

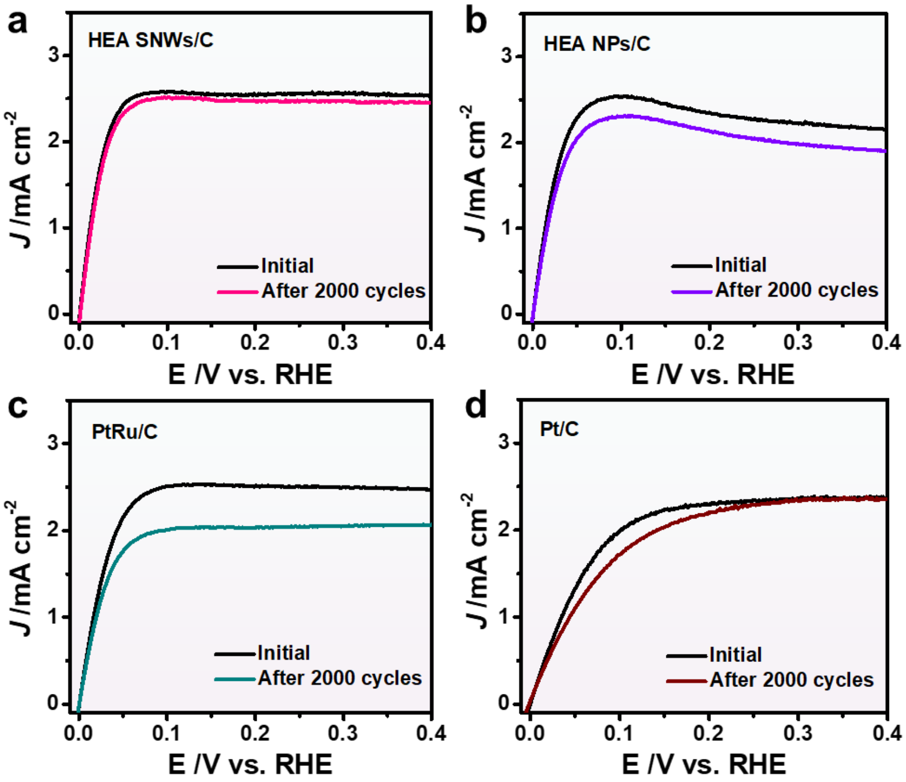

Supplementary Figure 17. Polarization curves of (a) HEA SNWs/C, (b) HEA NPs/C, (c) commercial PtRu/C, and (d) commercial Pt/C before and after 2000 cycles of ADTs between -0.1 and 0.4 V vs. RHE.

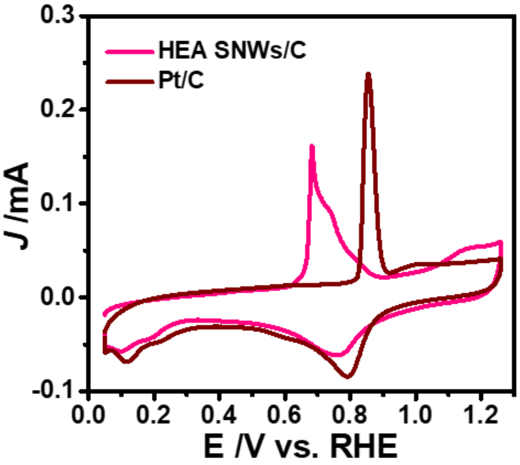

Supplementary Figure 18. CO-stripping curves of HEA SNWs/C and commercial Pt/C after 2000 cycles of ADT.

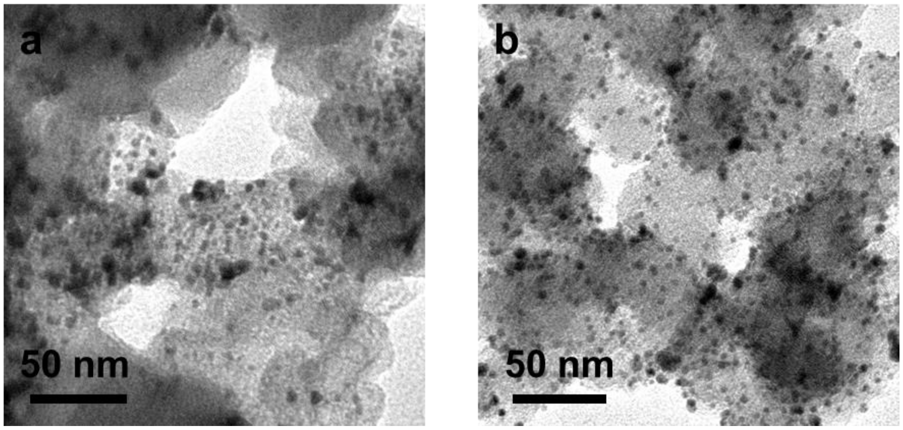

Supplementary Figure 19. TEM images of (a) commercial PtRu/C and (b) commercial Pt/C after 2000 cycles of ADT.

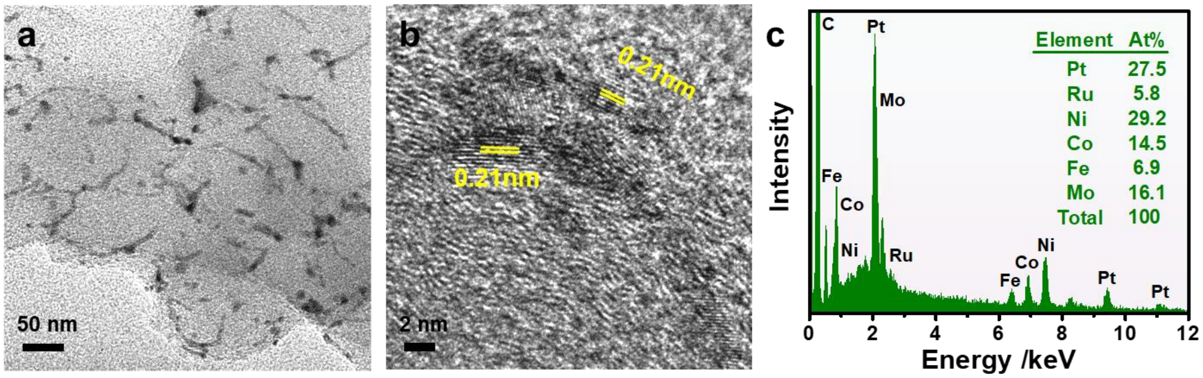

Supplementary Figure 20. (a) TEM image, (b) HRTEM image, and (c) SEM-EDS of HEA SNWs/C after 2000 cycles of ADT.

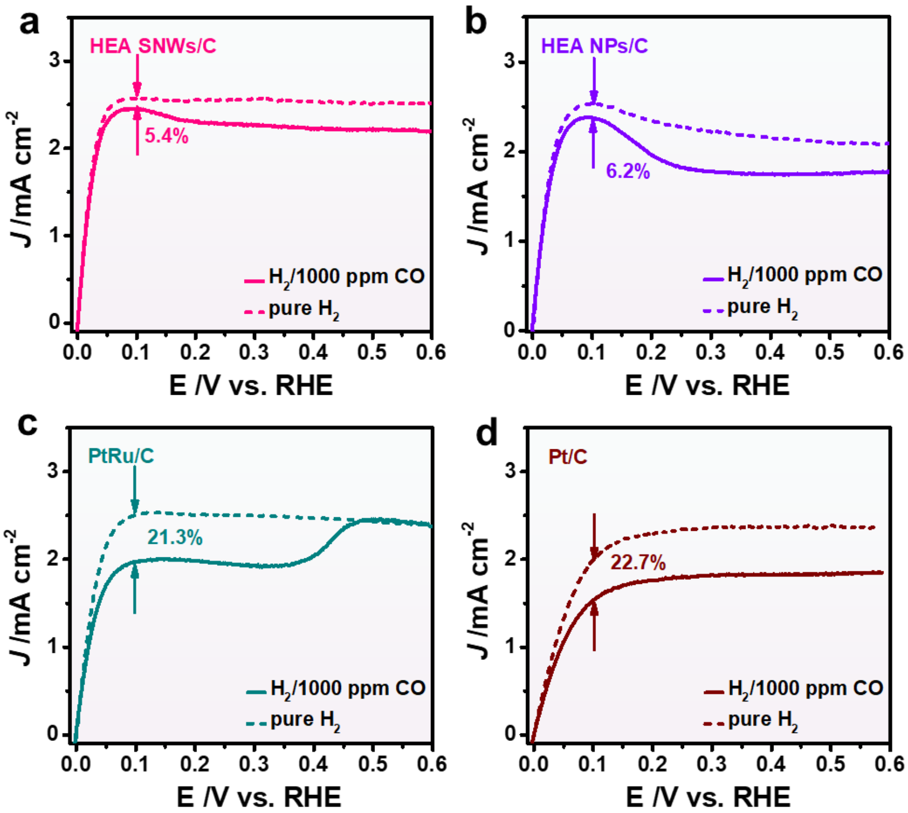

Supplementary Figure 21. Polarization curves of (a) HEA SNWs/C, (b) HEA NPs/C, (c) PtRu/C, and (d) Pt/C in 1000 ppm CO/H_2-saturated 0.1 M KOH.

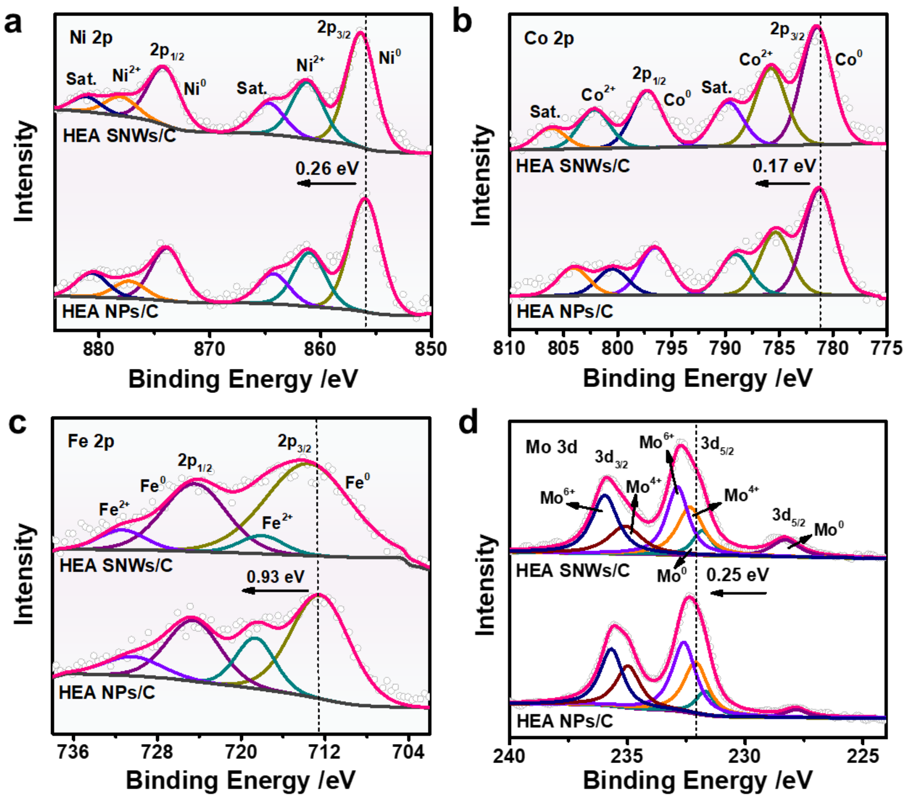

Supplementary Figure 22. XPS spectra of (a) Ni 2p, (b) Co 2p, (c) Fe 2p, and (d) Mo 3d for HEA SNWs/C and HEA NPs/C.

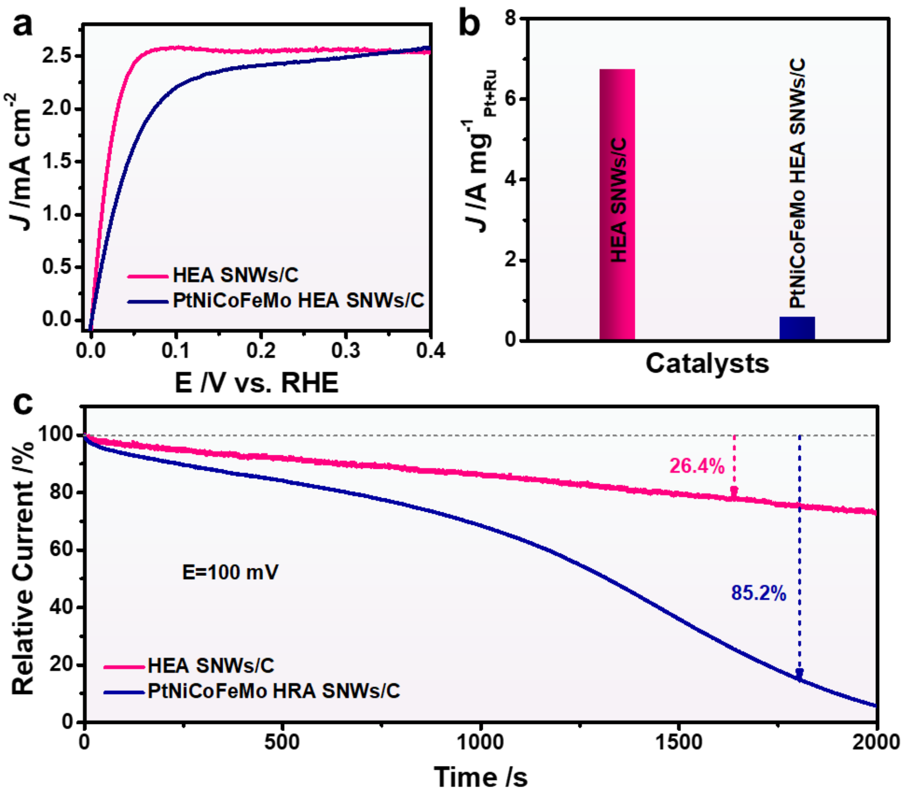

Supplementary Figure 23. (a) Polarization curves, (b) mass activities, and (c) relative current-time chronoamperometry responses of HEA SNWs/C (with Ru) and PtNiCoFeMo HEA SNWs/C (without Ru) in 1000 ppm CO/H_2-saturated 0.1 M KOH at 100 mV vs. RHE

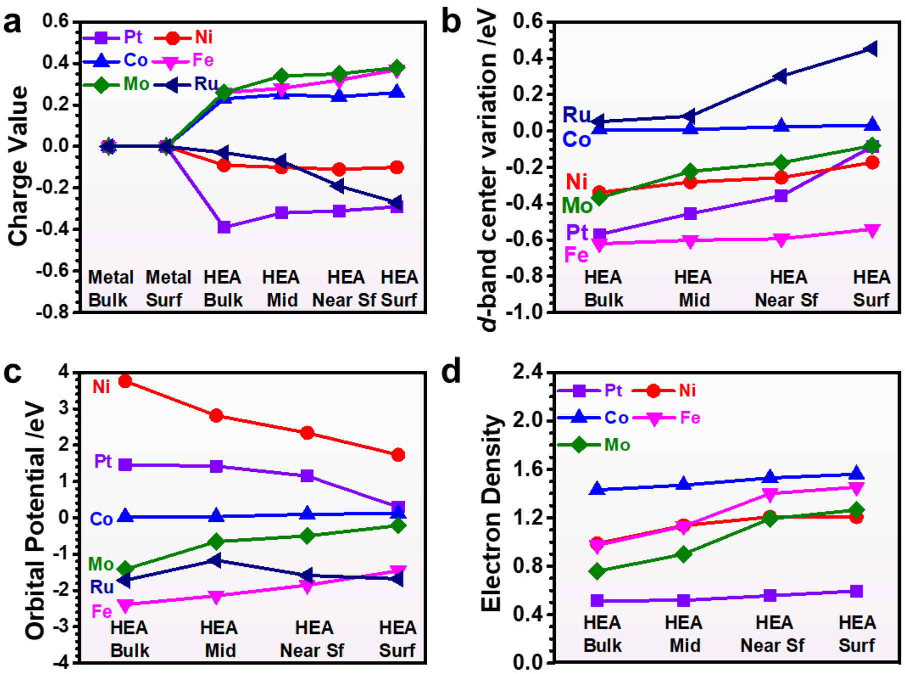

Supplementary Figure 24. Electronic structure characterizations of the HEA model. (a) The charge values of different elements. (b) The relative *d*-band change of different elements. (c) The orbital chemical potential of different elements. (d) The charge density near Fermi level of different elements.

Based on the DFT calculation result shown in Supplementary Figure 24, the additional illustrations are as follows.

With respect to HEA, the charge value of Ru displays a downshifting trend from the bulk to the surface, and Pt exhibits the most negative charge value among all the elements (Supplementary Figure 24a). The relatively negative charge values of Pt and Ru are consistent with the PDOS results, where Pt-5*d* and Ru-4*d* orbitals show a relatively deeper position with the electron-rich feature compared to other elements. We have then compared the relative *d*-band center of all the elements with the pristine metals (Supplementary Figure 24b). It is noted that the *d*-band centers of all the elements exhibit an upshifting trend from the bulk to the surface, which further verify the improved electroactivity for HOR. Moreover, Ru displays the lowest the orbital chemical potential compared to other elements (Supplementary Figure 24c), which may lead to the electron transfer trend from other elements to Ru, being consistent with the XPS results. Additionally, we have compared the electron density near the Fermi level to demonstrate electron transfer capability. As shown in Supplementary Figure 24d, all the metals display an upshifting trend of the electron density, which lowers the electron transfer barriers and increases the electron transfer efficiency towards the reaction intermediates, leading to the improved HOR performance on the HEA SNWs.

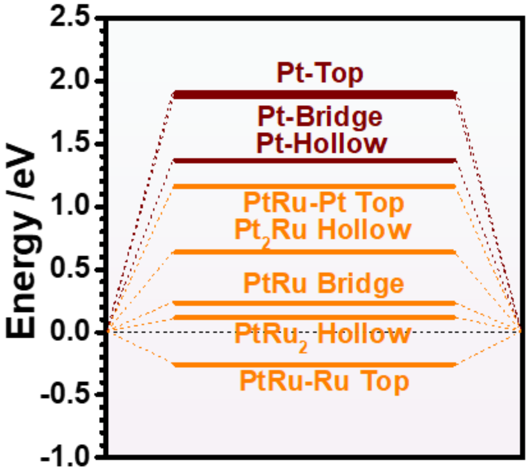

Supplementary Figure 25. The OH binding energies in Pt (111) and PtRu (111). On PtRu (111) surface, $PtRu_2$ hollow sites represent the hollow sites that constructed by one Pt and two Ru sites and the Pt_2Ru hollow sites represent the hollow sites constructed by two Pt and one Ru sites.

Supplementary Table 1. The loading amounts and ECSAs of HEA SNWs/C, HEA NPs/C, commercial PtRu/C, and commercial Pt/C.

Catalyst	Loading (μg_{Pt} cm^{-2})	Loading (μg_{Pt+Ru} cm^{-2})	ECSA (m^2 g_{Pt}^{-1})	ECSA (m^2 g_{Pt+Ru}^{-1})
**HEA SNWs/C**	7.7	8.8	87.1	75.3
**HEA NPs/C**	7.7	9.7	86.3	68.2
**PtRu/C**	7.7	11.7	66.7	43.7
**Pt/C**	7.7	7.7	70.2	70.2

Supplementary Table 2. The specific activities, mass activities, and exchange current at for HEA SNWs/C, HEA NPs/C, commercial PtRu/C, and commercial Pt/C at 50 mV vs. RHE.

Catalyst	$J_{m,\ 50\ mV}$ (A mg_{Pt}^{-1})	$J_{m,\ 50\ mV}$ (A mg_{Pt+Ru}^{-1})	$J_{s,\ 50\ mV}$ (mA cm^{-2})	I_0 (mA)	α
HEA SNWs/C	7.72	6.75	8.96	1.26	0.93
HEA NPs/C	2.98	2.37	3.47	0.84	0.68
PtRu/C	2.51	1.65	3.77	0.52	0.72
Pt/C	0.34	0.34	0.48	0.28	0.57

Supplementary Table 3. Summary of the reported catalysts for alkaline HOR.

Catalyst	Loading (μg_{NM^*} cm^{-2})	$J_{m,\ 50\ mV}$ (A mg_{NM}^{-1})	$J_{m,\ 50\ mV}$ (mA cm^{-2})	Ref.
**HEA SNWs/C**	**8.8**	**6.75**	**8.96**	**This work**
$Pt_{0.1}Ru_{0.9}$	~8	~1.9	~6.5	*1*
PtRu NWs	~20	0.6	2.2	*2*
PtRu/Mo_2C-TaC	13	0.403	0.28	*3*
Ru_7Ni_3/C	3.9	9.4	23.4	*4*
$RuNi_1$	8.8	2.7	/	*5*
Ru@TiO_2	230	~0.29	/	*6*
IrNi@Ir/C	10	1.12	/	*7*
PdCu/C-500 ℃	12.5	1.727	2.922	*8*
0.38 CeO_x-Pd/C	13	/	0.118	*9*
IrNi@PdIr/C	19.7	0.854	1.656	*10*

NM*: Noble metal.

References

1. Strmcnik, D. et al. Improving the hydrogen oxidation reaction rate by promotion of hydroxyl adsorption. *Nat. Chem.* **5**, 300-306 (2013).
2. Scofield, M. E. et al. Role of chemical composition in the enhanced catalytic activity of Pt-based alloyed ultrathin nanowires for the hydrogen oxidation reaction under alkaline conditions. *ACS Catal.* **6**, 3895-3908 (2016).
3. Hamo, E. R. et al. Carbide-supported PtRu catalysts for hydrogen oxidation reaction in alkaline electrolyte. *ACS Catal.* **11**, 932-947 (2021).
4. Xue, Y. et al. A highly-active, stable and low-cost platinum-free anode catalyst based on RuNi for hydroxide exchange membrane fuel cells. *Nat. Commun.* **11**, 5651 (2020).
5. Mao, J. et al. Isolated Ni atoms dispersed on Ru nanosheets: high-performance electrocatalysts toward hydrogen oxidation reaction. *Nano Lett.* **20**, 3442-3448 (2020).
6. Zhou, Y. et al. Lattice-confined Ru clusters with high CO tolerance and activity for the hydrogen oxidation reaction. *Nat. Catal.* **3**, 454-462 (2020).
7. Liu, D. et al. One-pot synthesis of IrNi@Ir core-shell nanoparticles as highly active hydrogen oxidation reaction electrocatalyst in alkaline electrolyte. *Nano Energy* **59**, 26-32 (2019).
8. Qiu, Y. et al. BCC-phased PdCu alloy as a highly active electrocatalyst for hydrogen oxidation in alkaline electrolytes. *J. Am. Chem. Soc.* **140**, 16580-16588 (2018).
9. Singh, R.K. et al. Synthesis of CeO_x-decorated Pd/C catalysts by controlled surface reactions for hydrogen oxidation in anion exchange membrane fuel cells. *Adv. Funct. Mater.* **30**, 2002087 (2020).
10. Qin, B. et al. A novel IrNi@PdIr/C core–shell electrocatalyst with enhanced activity and durability for the hydrogen oxidation reaction in alkaline anion exchange membrane fuel cells. *Nanoscale* **10**, 4872-4881 (2018).
